# *RGN1* controls grain number and shapes panicle architecture in rice

**DOI:** 10.1111/pbi.13702

**Published:** 2021-09-22

**Authors:** Gangling Li, Bingxia Xu, Yanpei Zhang, Yawen Xu, Najeeb Ullah Khan, Jianyin Xie, Xingming Sun, Haifeng Guo, Zhenyuan Wu, Xueqiang Wang, Hongliang Zhang, Jinjie Li, Jianlong Xu, Wensheng Wang, Zhanying Zhang, Zichao Li

**Affiliations:** ^1^ State Key Laboratory of Agrobiotechnology/Beijing Key Laboratory of Crop Genetic Improvement College of Agronomy and Biotechnology China Agricultural University Beijing China; ^2^ Institute of Crop Sciences Chinese Academy of Agricultural Sciences Beijing China

**Keywords:** germplasm, *Regulator of Grain Number1*, grain number per panicle, MYB transcription factor

## Abstract

Yield in rice is determined mainly by panicle architecture. Using map‐based cloning, we identified an R2R3 MYB transcription factor REGULATOR OF GRAIN NUMBER1 (RGN1) affecting grain number and panicle architecture. Mutation of *RGN1* caused an absence of lateral grains on secondary branches. We demonstrated that *RGN1* controls lateral grain formation by regulation of *LONELY GUY* (*LOG*) expression, thus controlling grain number and shaping panicle architecture. A novel favourable allele, *RGN1* ^C^, derived from the Or‐I group in wild rice affected panicle architecture by means longer panicles. Identification of *RGN1* provides a theoretical basis for understanding the molecular mechanism of lateral grain formation in rice; *RGN1* will be an important gene resource for molecular breeding for higher yield.

## Introduction

Rice is a staple food for almost half of the world population (Sasaki and Burr, 2000). Grain yield is determined by plant architecture which includes plant height, tiller number and angle, and panicle architecture (Jiao *et al*., 2010), which in turn comprises grain number, grain size and panicle size including branches (Tabuchi *et al*., 2011).

Many genes affecting panicle development in rice and other grasses have been identified (Ikeda *et al*., 2007; Ikeda‐Kawakatsu *et al*., 2012; Ishikawa *et al*., 2005; Jiang *et al*., 2013; Koumoto *et al*., 2013; Kwon *et al*., 2012; Li *et al*., 2013; McSteen, 2009; McSteen *et al*., 2007; Raman *et al*., 2008; Spinelli *et al*., 2011; Takeda *et al*., 2003; Tanaka *et al*., 2015; Wu *et al*., 2016; Yao *et al*., 2019; Yoshida *et al*., 2013; Zhou *et al*., 2013). Most of them are transcription factors or transcriptional regulators. For example, *FRIZZY PANICLE* (*FZP*) encodes an ERF transcription factor containing an AP2 domain, which can prevent axillary meristem (AM) formation, but can also promote the formation of flower meristems (Komatsu *et al*., 2003b). Researchers have found that an 18‐bp insertion at 5.3 kilobase (kb) upstream of *FZP* leads to decreased expression of *FZP* and a consequent increase in yield (Bai *et al*., 2017). *LAX PANICLE1* (*LAX1*) encodes a bHLH transcription factor that regulates initiation of AM (Komatsu *et al*., 2003a). *LAX1* mRNA is specifically expressed in the boundary region between the point of AM initiation and shoot apical meristem; LAX1 protein is subsequently trafficked towards the AM where it enhances cell proliferation (Oikawa and Kyozuka, 2009). Mutation of *LAX1* caused the absence of lateral grains from secondary branches (SBs). Mutation of *LAX1* homologs *BARREN STALK1* (*BA1*) in maize (Gallavotti *et al*., 2004), and *REGULATOR OF AXILLARY MERISTEM FORMATION* (*ROX*) in Arabidopsis showed similar defects in panicle and shoot branching respectively (Yang *et al*., 2012). Recent studies in maize showed that *BA1* was involved in auxin signalling during reproductive AM initiation and was directly regulated by BARREN INFLORESCENCE1 (BIF1) and BARREN INFLORESCENCE4 (BIF4) (Galli *et al*., 2015). *LAX1* in rice can also function along with *MONOCULM1* (*MOC1*) which encodes a transcriptional regulator of the GRAS family. Disruption of *MOC1* in rice, or homologous genes *LATERAL SUPPRESSOR* (*LAS*) in Arabidopsis and *Lateral suppressor* (*Ls*) in tomato (*Solanum lycopersicum*), leads to a lack of shoot branching (Greb *et al*., 2003; Li *et al*., 2003; Schumacher *et al*., 1999). In rice, *LAX2*/*GNP4* genetically interacts with both *LAX1* and *MOC1* which encodes a nuclear protein with a conserved RAWUL domain. Loss of function of *LAX2/GNP4* affects both vegetative and reproductive branching (Tabuchi *et al*., 2011; Zhang *et al*., 2011).

Plant hormones also play important role in regulating branch and spikelet number in rice. *GRAIN NUMBER 1a* (*Gn1a*), the first major QTL implicated in to grain number per panicle explained 44% of the phenotypic variation in a biparental cross (Ashikari *et al*., 2005). *Gn1a* encodes cytokinin oxidase OsCKX2 that degrades cytokinin. *LONELY GUY* (*LOG*) encoding a cytokinin activating enzyme with opposite effect to *Gn1a* plays an important role in the final step of cytokinin biosynthesis (Kurakawa *et al*., 2007). Meristematic activity in a *log* mutant could not be maintained and led to early termination of panicle development, causing a shorter panicle and reduced number of panicle branches.

Gain‐of‐function mutant *exb1‐D* in Arabidopsis displays a dramatically increased number of branches that resulted from over‐expression of *EXCESSIVE BRANCHES1* (*EXB1*) (Guo *et al*., 2015). *EXB1*, encoding WRKY transcription factor WRKY71 with transactivation activity, positively regulates *REGULATOR OF AXILLARY MERISTEMS* (*RAX*) genes at the transcriptional level. *RAX* genes encode R2R3 MYB family transcription factors that are also involved in shoot branching (Keller *et al*., 2006; Muller *et al*., 2006; Schmitz *et al*., 2002). Double and triple mutants show that *RAX1* functions partially redundantly with *ROX* and *LAS* in regulating AM formation (Yang *et al*., 2012). Orthologous *RAX* genes in other dicots species such as *BLIND* (*BL*) in tomato and *CaBLIND* (*CaBL*) in pepper (*Capsicum annuum*) also have a role in regulating AM initiation (Jeifetz *et al*., 2011; Schmitz *et al*., 2002), but the homolog in monocots has not been identified (McSteen, 2009). Hence, the role of MYB transcription factors in regulating branching in monocots remains to be elucidated.

In this study, *REGULATOR OF GRAIN NUMBER1* (*RGN1*) was identified by map‐based cloning using segregating population constructed by rice germplasm resources with different grain number per panicle. *RGN1* encodes an R2R3 MYB transcription factor, with high similarity to RAX in Arabidopsis. *RGN1* plays a significant role in regulating panicle architecture and grain number together with *LOG*. Plants with the loss‐of‐function mutant allele *rgn1* displayed highly decreased grain number. Elite haplotype *RGN1* ^C^ for panicle length identified in natural germplasm has potential to improve the yield of rice.

## Results

### Phenotypic characterization of NIL‐*RGN1* and NIL‐*rgn1*

**Figure 1:**
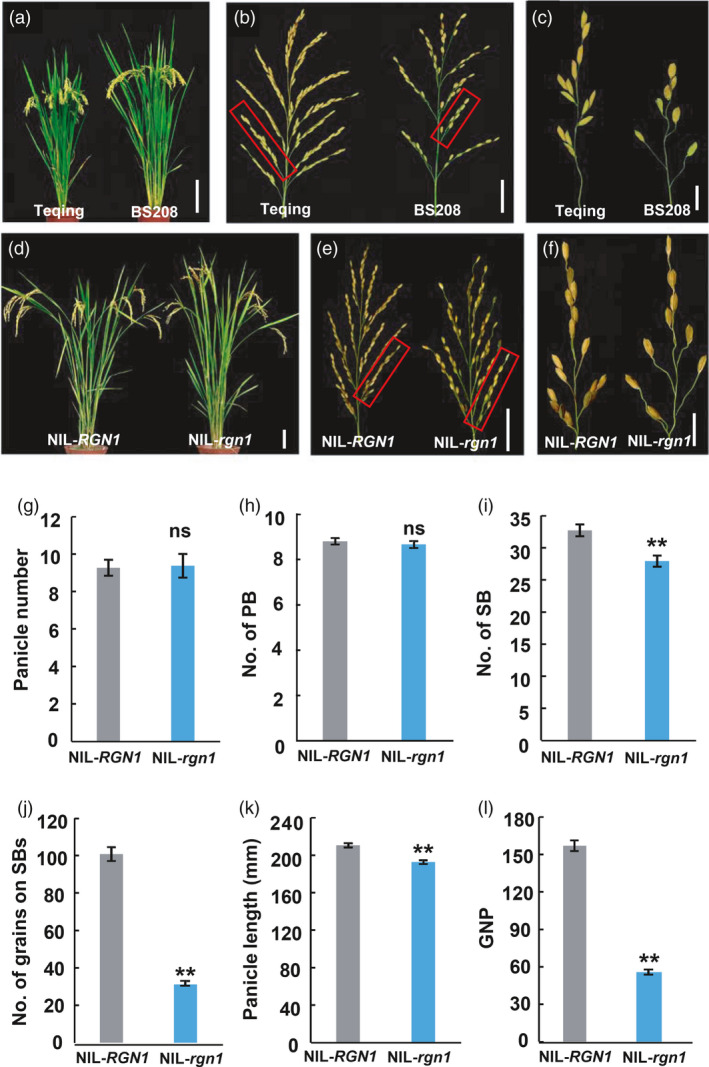
Phenotypic characterization of Teqing, BS208 and *RGN1*/*rgn1* NILs. (a–c) Plant architecture (a), panicles (b) and primary branches (c) from (b) within red boxes of Teqing and BS208. (d–f) Plant architecture (d), panicles (e), primary branches (f) from (e) within red boxes of NIL‐*RGN1* and NIL‐*rgn1*. (g–l) Statistical comparisons of panicle number (g), primary branches number (h), secondary branches number (i), grain number on secondary branches (j), panicle length (k) and grain number per panicle (l) of NIL‐*RGN1* and NIL‐*rgn1*. PB, primary branch; SB, secondary branch; GNP, grain number per panicle. Values are means ± SEM (*n* = 12). **, *P* < 0.01, ns, no significant difference. Two‐tailed student’s *t*‐tests. Scale bars, 20 cm for (a, d); 5 cm for (b, e); 2 cm for (c, f).

We undertook a germplasm screening program to explore and dissect genetic networks involved in regulation of panicle architecture in rice. A *japonica* variety named BS208 showed a highly abnormal panicle branching pattern, with reduced numbers of lateral grains on SBs (Figure 1a–c and Figure S1). To determine the genetic mechanism underlying the abnormal phenotype, we crossed BS208 with *indicia* variety Teqing (TQ). F_1_ plants had normal panicle architecture, and the F_2_ population segregated 338 normal: 117 abnormal panicle phenotype indicative of segregation at a single locus (χ^2^ = 0.124; *P*_1d.f__._ > 0.9), which was named *RGN1*. To detail the effects of *RGN1* on panicle development, a pair of near‐isogenic lines (NIL) was developed with the *RGN1* allele from TQ and *rgn1* allele from BS208, namely NIL‐*RGN1* and NIL‐*rgn1* (Figure S2). The NILs had significant differences in panicle phenotype (Figure 1d–f). The numbers of panicles and primary branches (PBs) in the NILs were similar (Figure 1g,h), but the numbers of SBs, number of lateral grains on the SBs and panicle length in NIL‐*rgn1* were greatly reduced (Figure 1i–k). Thus, the grain number per panicle in NIL‐*rgn1* was decreased (Figure 1l). Although the grain size and grain weight were increased in NIL‐*rgn1,* the yield per plant was significantly decreased compared with NIL‐*RGN1* (Figure S3). These overall results indicated that *RGN1* was involved in panicle architecture and the formation of lateral grains on secondary branches.

### Map‐based cloning of *RGN1*

**Figure 2:**
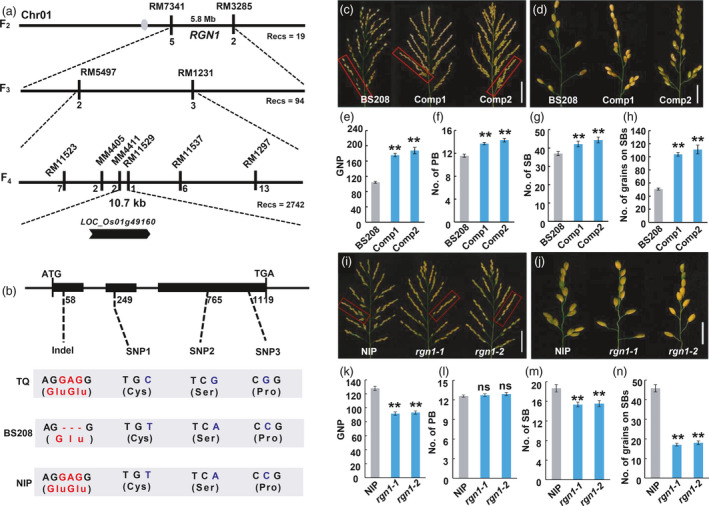
Mapping and validation of *RGN1*. (a) Summary of *RGN1* mapping. Recs, numbers of plants with the recessive phenotype; numbers below the markers indicate numbers of recombinant individuals. (b) Comparison of DNA sequence of *LOC_Os01g49160* for TQ, BS208 and NIP. The numbers below the schematic of the gene structure indicate the positions of the start codon (ATG), the stop codon (TGA) and polymorphic nucleotide(s). The first nucleotide of start codon is as No. 1. (c,d) Panicle architectures (c), primary branches (d) from (c) within red boxes of BS208 and complementation lines Comp1 and Comp2. (e–h) Statistical comparisons of grain number per‐panicle (e), primary branch number (f), secondary branch number (g) and number of grains on secondary branches (h) of BS208, Comp1 and Comp2 plants. (i–j) Panicle architectures (i), primary branches (j) from (i) within red boxes of NIP, *rgn1‐1* and *rgn1‐2* plants. (k–n) Statistical results for grain number per panicle (k), primary branch number (l), second branches number (m) and number of grains on secondary branch (n) of NIP, *rgn1‐1* and *rgn1‐2* plants. Values are means ± SEM, (*n* = 12). **P < 0.01, ns, no significant difference. Two‐tailed student’s *t*‐tests. Scale bars, 5 cm for (c, i); 2 cm for (d,j).

Heterozygous lines were selected to isolate the candidate gene for *RGN1* by map‐based cloning. The *RGN1* locus was initially mapped to the long arm of chromosome 1 flanked by the SSR markers RM7341 and RM3285 by analysis of F_2_ plants with the recessive phenotype from cross BS208 ×TQ. The locus was then fine mapped to a 10.7 kb region flanked by SSR markers MM4411 and RM11529 using an F_3:4_ population (Figure 2a). According to the Rice Genome Annotation Project (RGAP) only one open reading frame, namely *LOC_Os01g49160,* was predicted within this genomic region (Figure 2a). *LOC_Os01g49160* encodes an R2R3 MYB transcription factor containing three exons and two introns. Comparison of the genomic DNA sequences showed three synonymous SNP and one indel (GAG 58–60) difference between TQ and BS208, and the indel (GAG 58–60) caused a single amino acid deletion in the conserved R2R3 domain (Figure 2b). We also noted this indel (GAG 58–60) difference between BS208 and Nipponbare (NIP) in the 10.7 kb region (Figure 2b). These findings indicated that *LOC_Os01g49160* was a strong candidate for *RGN1*.

To further confirm that *LOC_Os01g49160* was the correct gene, we performed a genetic complementation test in which the coding sequence (CDS) of *LOC_Os01g49160* from NIP driven by its native promoter was introduced into BS208 by *Agrobacterium tumefaciens*‐mediated transformation. Positive complementary lines (Comp1 and Comp2) regenerated lateral grains on the SBs (Figure 2c,d and Figure S4). Quantitative analysis of Comp1 and Comp2 showed that the numbers of primary and secondary branches, panicle length and grain number per panicle were significantly increased compared with BS208 (Figure 2e–h). In addition, we produced two homozygous mutants of *LOC_Os01g49160* named *rgn1‐1* and *rgn1‐2* in NIP background by a CRISPR/Cas9 approach. Lines *rgn1‐1* and *rgn1‐2* harboured 2 bp and 5 bp deletions, respectively, in *LOC_Os01g49160* CDS (Figure S5). The absence of lateral grains at SBs in both mutants caused a panicle phenotype that was similar to of BS208 (Figure 2i–n). We concluded that *LOC_Os01g49160* was responsible for shaping panicle architecture in rice, and the locus is hereafter referred to as *RGN1*.

Next, an over‐expression construct was generated and introduced into NIP. Positive transgenic lines showed no significant differences in branch number and grain number compared to the wild type (Figure S6). We also obtained a T‐DNA insertion mutant, *rgn1‐D*, with an activation tag inserted into the promoter region in which allowing the expression level of the *RGN1* allele to increase (Figure S7a–d). There was no obvious difference in panicle architecture between the *rgn1‐D* mutant and wild type cultivar Dongjin (Figure S7e–i). These results indicated that the increased expression level of *RGN1* had no positive influence on panicle development.

### *RGN1* encodes an R2R3‐MYB transcription factor

Although the rice annotation database predicted that *RGN1* encoded an R2R3 MYB transcription factor, its function is unclear. From protein blast results, we found that the RGN1 protein was a homolog of RAX in Arabidopsis (Figure S8) that functioned redundantly in control of shoot branching (Keller *et al*., 2006; Muller *et al*., 2006). The functions of *RAX* genes were known to be conserved in dicots, but until now homologs of *RAX* genes in monocots had not been identified. A phylogenetic analysis showed that protein homologs of RGN1 in the *Poaceae* were highly conserved compared with other species (Figure S9).

**Figure 3:**
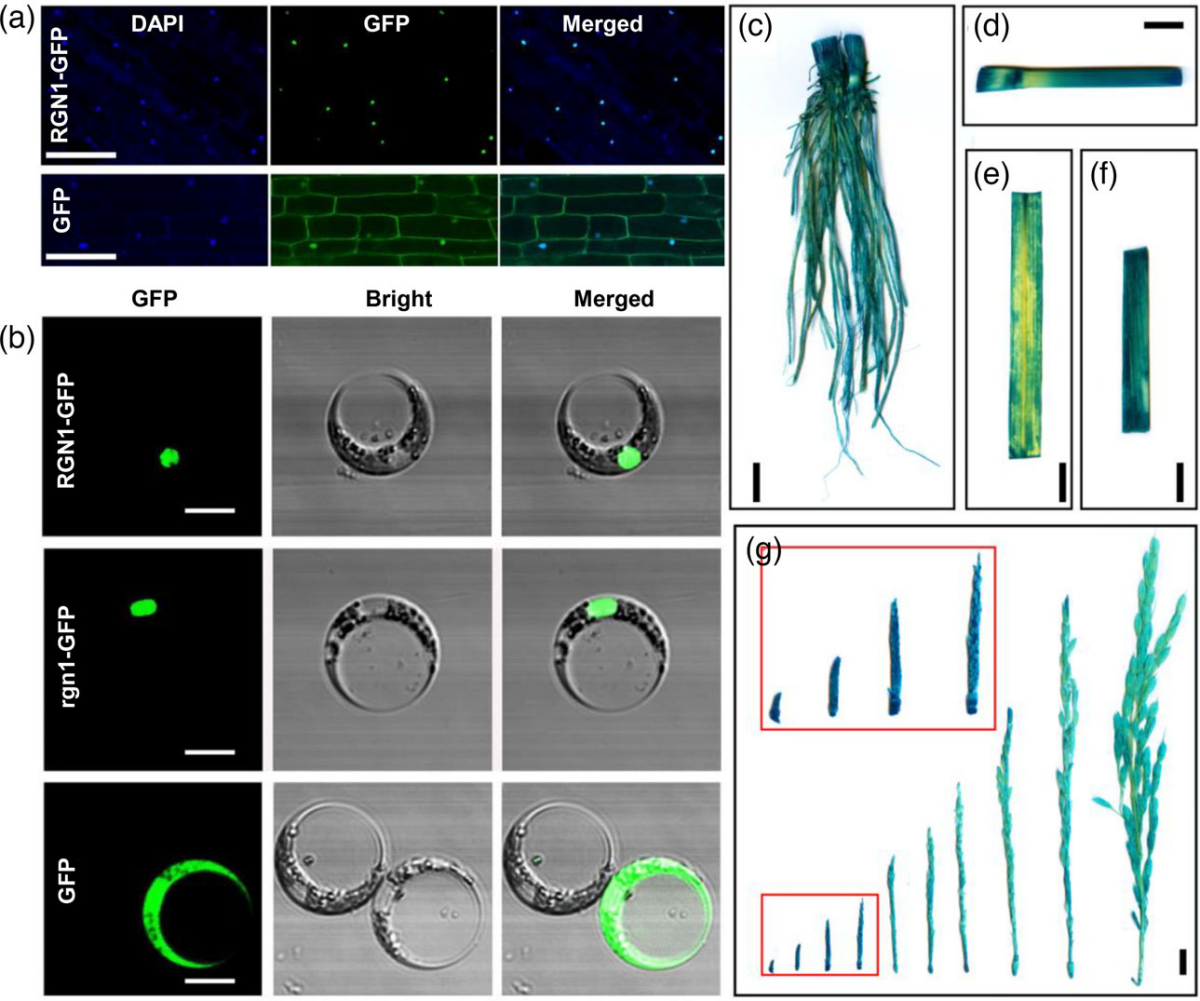
Expression pattern of RGN1. (a) Subcellular localization of RGN1‐GFP in root tips of transgenic plants. (b) Subcellular localization of RGN1‐GFP and rgn1‐GFP fusion protein in rice protoplast cells. Empty vector pSuper1300‐GFP was used as control in (a) and (b). (c–g) GUS staining of tissues from *ProRGN1:GUS* transgenic plants. Root (c), stem (d), leaf (e), leaf sheath (f), panicles at different stages (g). Scale bars, 10 μm for (a); 20 μm for (b); 1 cm for (c–g).

We generated transgenic plants with a 35S:RGN1‐green fluorescent protein (GFP) signal to determine the subcellular localization of RGN1. Green fluorescence was detected in the nuclei of root tip cells (Figure 3a). Transient expression experiments in rice protoplasts showed similar results (Figure 3b). These results were consistent with RGN1 having a role as a transcription factor. We also noted that the mutant allele in BS208 did not change the subcellular location of rgn1 protein (Figure 3b). To explore the expression pattern of *RGN1*, we generated *ProRGN1: GUS* transgenic plants and GUS activity was detected in various organs, especially in young panicles (Figure 3c–g), and consistent with its function in regulating panicle development. We also found similar results by quantitative RT‐PCR analyses. Quantitative RT‐PCR analyses showed that *RGN1* was expressed in roots, stems, leaves, leaf sheaths and young panicles at different developmental stages (Figure S10).

### RGN1 activates expression of *LOG* to affect panicle architecture

**Figure 4:**
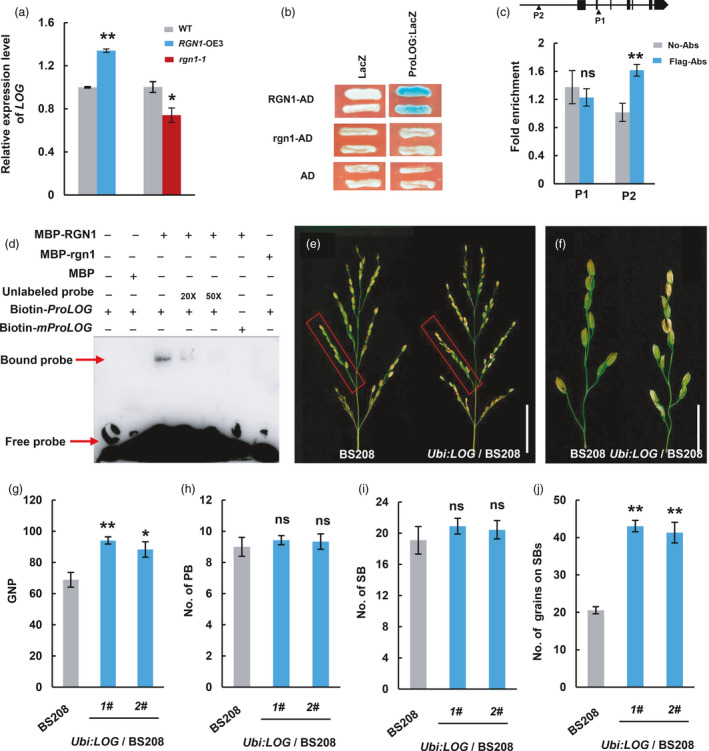
RGN1 controls *LOG* expression to regulate the formation of lateral grains on secondary branches. (a) Expression levels of *LOG* in NIP compared to *rgn1‐1* and *RGN1*‐OE3 plants. Values are means ± SEM, (*n* = 3, each with three technical repeats). (b) Yeast one‐hybrid assays demonstrate that RGN1 binds to the *LOG* promoter (*ProLOG*). (c) Quantitative ChIP‐PCR indicates that RGN1 binds to the MS188‐binding motif (P2 region) from the *LOG* promoter *in vivo*. Values are means ± SEM, (*n* = 3). (d) Electrophoretic mobility shift assay shows that RGN1 binds to the MS188‐binding motif from the *LOG* promoter *in vitro*. m*ProLOG*, *LOG* promoter containing mutated MS188 binding motif. (e,f) Phenotype of BS208 was partially rescued by *LOG* overexpression: panicles architecture (e), primary branches (f) from (e) within red boxes of BS208 and *Ubi:LOG*/BS208 transgenic plant. Scale bars, 5 cm for (e); 2 cm for (f). (g–j) Comparisons of grain number per panicle (g), primary branch number (h), secondary branches number (i), number of grains on secondary branches (j) between BS208 and *Ubi:LOG/BS208* transgenic plants. Values are means ± SEM (*n* = 10). *P < 0.05, **P < 0.01, ns, no significant difference. Two‐tailed student’s *t*‐tests.

Previous studies showed that cytokinin (CK) was an essential regulators of inflorescence architecture (Han *et al*., 2014). Two cytokinin related genes in rice *Gn1a* (*OsCKX2*) and *LOG* were reported to be involved in panicle development (Ashikari *et al*., 2005; Kurakawa *et al*., 2007). To determine the relationship of *Gn1a* and *LOG* with *RGN1*, we first examined the expression levels of *Gn1a* and *LOG* in NIP and *rgn1‐1*, NIL‐*RGN1* and NIL‐*rgn1* plants. *LOG* expression was down‐regulated in *rgn1‐1* and NIL‐*rgn1* compared with NIP and NIL‐*RGN1* respectively (Figure 4a and Figure S11a), whereas *Gn1a* expression was up‐regulated (Figure S11b,c). Additionally, yeast one‐hybrid assays (Y1H) showed that RGN1 could not bind to the promoter of *Gn1a* (Figure S11d). We next checked *LOG* expression in *RGN1*‐OE3 plants and found that it was up‐regulated (Figure 4a). These results suggested that *RGN1* might participate in cytokinin metabolism by regulating *LOG* expression. Yeast one‐hybrid assays showed that RGN1 bound directly to the promoter region of *LOG* (Figure 4b). As the binding sites of RGN1 and its homologs in rice had not been identified, we searched the Plant Transcription Factor Database for RGN1 homologs in Arabidopsis and found that the binding motif of AtMYB103/MS188 had been identified by DNA affinity purification sequencing (DAP‐seq) (Jin *et al*., 2017; O'Malley *et al*., 2016) (Figure S11e). Then, we screened the *LOG* promoter and found one MS188‐binding motif. EMSA showed that RGN1 could bind to the MS188‐binding motif of the *LOG* promoter, whereas rgn1 could not. Quantitative chromatin immunoprecipitation PCR (qChIP‐PCR) assays indicated that the DNA fragment containing the MS188‐binding site from the *LOG* promoter was enriched by affinity‐purified anti‐Flag antibodies (Figure 4c–d). These experiments thus showed that RGN1 could regulate *LOG* both *in vitro* and *in vivo*.

Next, the contents of bioactive CKs in young panicles of NIL‐*RGN1* and NIL‐*rgn1* were compared to determine the effect of *RGN1* on endogenous CK contents. Compared with NIL‐*RGN1*, the bioactive CKs (IP and cZ) contents in NIL‐*rgn1* were significantly lower (Figure S11f). In addition, over‐expression of *LOG* in BS208 partially rescued the absence of lateral grains in secondary branches (Figure 4e–j and Figure S11g). These findings indicated that RGN1 regulates *LOG* to control lateral grain development thus determining panicle architecture.

### Elite allele *RGN1* ^C^ for panicle length was identified from natural germplasm of *O*. *sativa* and *O*. *rufipogon*

**Figure 5:**
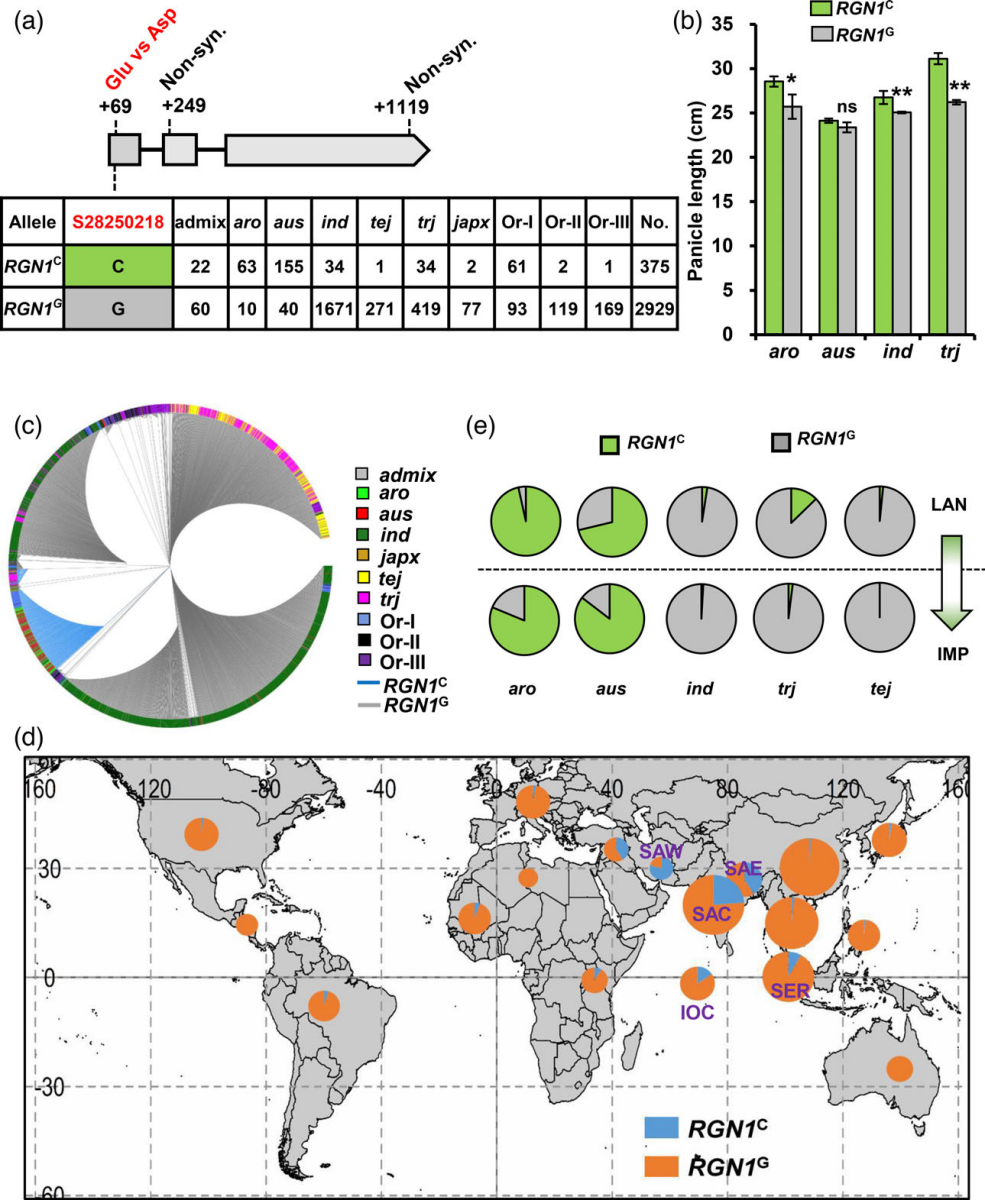
Variation of *RGN1* in germplasm. (a) Haplotypes of *RGN1* based on SNP S28250218. (b) Statistical comparison for panicle length in accessions containing the *RGN1* ^C^ and *RGN1* ^G^ alleles. Data are means ± SEM, *n* = 44 and 7 (*aro*), 132 and 30 (*aus*), 23 and 969 (*ind*), 29 and 247 (*trj*) for *RGN1* ^C^ and *RGN1* ^G^ alleles respectively. *P < 0.05; **P < 0.01; Two‐tailed student’s *t*‐tests. (c) Phylogram of *RGN1* generated from 2848 cultivated and 446 wild rice accessions. (d) Geographical distribution of the accessions containing *RGN1* ^C^ or *RGN1* ^G^. SAW (South Asia‐West, including Afghanistan and Pakistan), SAE (South Asia‐East, including Bangladesh and Bhutan), SAC (South Asia‐Central, including India and Nepal), SER (SEA islands, including Brunei, Indonesia, Malaysia and the Philippines), IOC (Indian Ocean, including Madagascar and Sri Lanka). (e) Frequencies of *RGN1* ^C^ and *RGN1* ^G^ in landraces (LAN) and improved varieties (IMP).

An investigation of sequence variation at the *RGN1* locus in 2858 cultivated and 446 wild rice accessions encompassing taxonomic groups admix, *aromatic* (*aro*), *aus*, *indica* (*ind*), temperate *japonica* (*tej*), tropical *japonica* (*trj*) and three types *O*. *rufipogon* species (Or‐I, Or‐II and Or‐III) identified three SNPs (S28250218, S28250398 and S28251268) (Data S1). Only S28250218 was non‐synonymous and resulted in a single amino acid change (Glu vs. Asp) in the conserved R2R3 DNA‐binding domain of RGN1 (Figure 5a, Data S1 and Figure S8). However, the GAG 58‐60 difference Indel between TQ and BS208 was not identified, indicating that the rare *RGN1* ^BS208^ allele could be a spontaneous mutant unique to BS208. Based on S28250218, the accessions were divided into two haplotypes namely Hap‐*RGN1* ^C^ and Hap‐*RGN1* ^G^. Accessions containing the *RGN1* ^C^ allele exhibited longer panicles than accessions containing the *RGN1* ^G^ allele (Figure 5b). Expression of *LOG* was higher in accessions containing *RGN1* ^C^ than accessions containing *RGN1* ^G^ (Figure S12a). Transient expression of *LOG* was also higher in rice protoplasts expressing RGN1^C^ than in protoplasts expressing RGN1^G^ or rgn1 (Figure S12b). The phylogenetic tree of *RGN1* base on 13 SNPs in the genomic region including a 2 kb promoter indicated that all wild and cultivated accessions with *RGN1* ^C^ had high similarity (Figure 5c). The *RGN1* ^C^ allele in wild rice was very frequent in Or‐I wild rice accessions (61 of 64, 95.3%) and was the most common allele in the overall Or‐I group (61 of 154 accessions, 39.6%). It was also common in *aro* (63 of 73 accessions, 86.3%) and *aus* (155 of 195 accessions, 79.5%), but was a minor allele in *ind* (34 of 1705, 2.0%) and *trj* (34 of 453, 7.5%) and an extremely rare allele in *tej* (1 of 272, 0.4%) (Figure 5a). These results suggest that the *RGN1* ^C^ allele in both wild and cultivated rice might originate in wild rice Or‐I prior to domestication.

Although *RGN1* ^C^ was found in wild and cultivated accessions distributed worldwide, it was more frequent among accessions from the South Asia‐Central (SAC), South Asia‐East (SAE), SEA Islands (SER), South Asia‐West (SAW) and Indian Ocean (IOC) region (Figure 5d). As the proportions of accessions containing *RGN1* ^C^ were lower in both landraces (LAN) and improved varieties (IMP) within the *ind*, *trj* and *tej* groups, there is considerable potential for molecular breeding to improve panicle architecture in these groups (Figure 5e). Further study of the function of *RGN1* ^C^ may provide clearer evidences for its utilization in yield improvement.

## Discussion

Branching is an iconic characteristic that plant obtained through body plan evolution (Graham *et al*., 2000). Branching in rice causes the formation of tillers and panicle branches. The first step in branching is AM initiation (Stirnberg *et al*., 2002). Initiation of AM in Arabidopsis requires the participation of at least three groups of transcription factors: *ROX*, *LAS* and *RAX1* (Yang *et al*., 2012). The *ROX* homolog in rice is *LAX1*, and the *LAS* homolog is *MOC1*. The *RAX* homolog was previously unknown. Here, we demonstrated that *RGN1* encodes an R2R3 MYB transcription factor that is homologous to *RAX1* and controls lateral grains formation.

**Figure 6:**
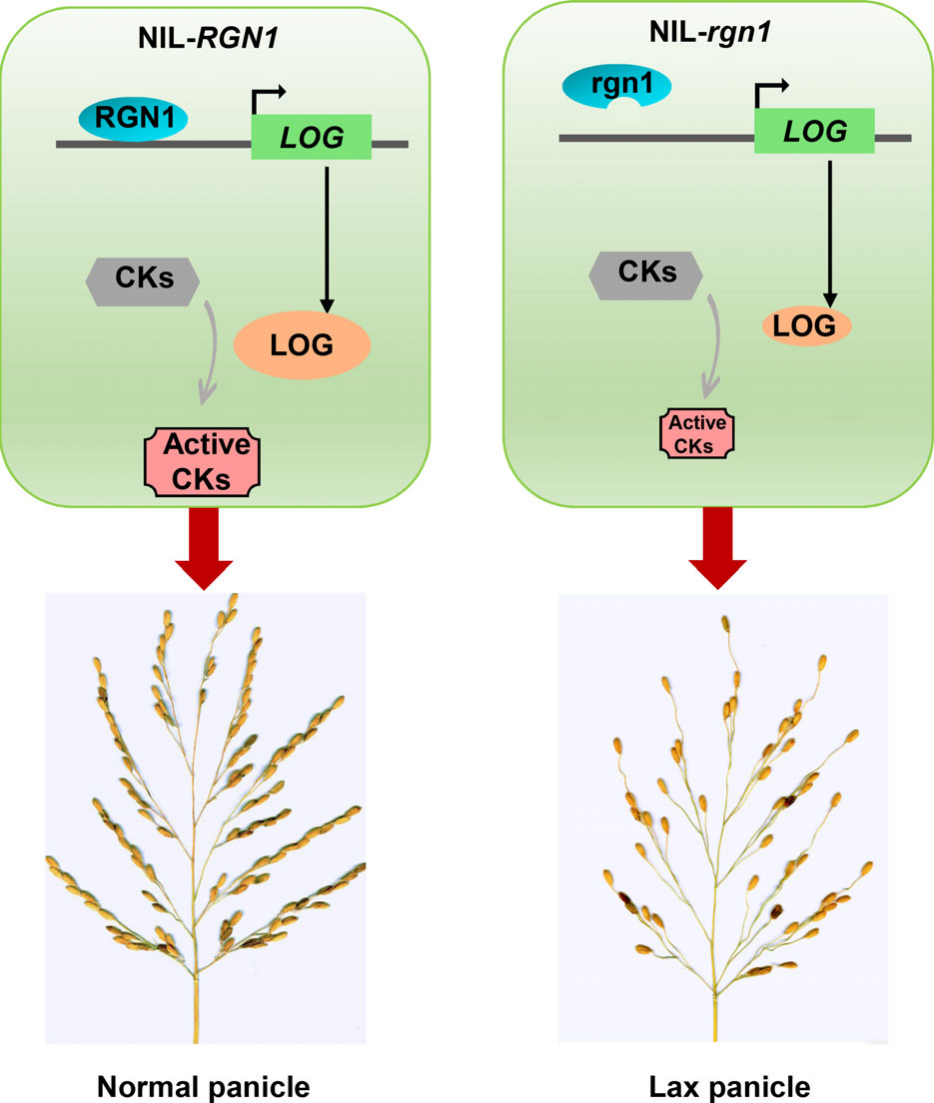
Model showing the difference in AM formation between NIL‐*RGN1* and NIL‐*rgn1*. Grey arrows are previously known pathways; black arrows indicate direct regulations demonstrated in the current work.

Considering all results from this study, we proposed a working model explaining the difference in panicle development between NIL‐*RGN1* and NIL‐*rgn1* (Figure 6). The RGN1 protein in NIL‐*RGN1* directly binds to the *LOG* promoter to positively regulate *LOG* expression. LOG transforms inactive CKs to active forms that amplify the CK signalling pathway (Kurakawa *et al*., 2007). The amplified CK signalling causes NIL‐*RGN1* to develop a normal panicle. The contrasting situation in NIL‐*rgn1* is that the rgn1 protein fails to bind to the *LOG* promoter, thus preventing amplification of CK signalling. Unamplified CK signalling causes NIL‐*rgn1* to develop a lax panicle.

Elevated expression of *RGN1* had no obvious effect on panicle branch formation (Figures S6 and S7). When *LAX1* or *LAX2/GNP4* was over‐expressed, the effect on panicle branching was similar to that of *RGN1* (Komatsu *et al*., 2003a; Zhang *et al*., 2018). However, mutations in *RGN1*, *LAX1 or LAX2/GNP4* strongly suppress lateral grain formation, implying that *RGN1*, *LAX1* and *LAX2/GNP4* are essential for determination of panicle architecture. These indicated that the vital function of these genes in regulating panicle architecture. Coincidently, we found the *RGN1* ^BS208^ allele only in accession BS208 despite and search of a large panel of wild and cultivated rice accessions, providing evidence that *RGN1* has an indispensable role in maintaining basic panicle architecture. It could be considered an indispensable house‐keeping or core‐genes that are essential for plant growth, development and reproduction (Wang *et al*., 2018).

We also isolated a novel *RGN1* ^C^ allele, in which SNP S28250218 caused an amino acid change (Glu vs. Asp) in the conserved DNA‐binding domain. The increased panicle size in accessions with *RGN1* ^C^ may be caused by the enhanced regulation of genes downstream of RGN1^C^. This variation (from Glu in RGN1^G^ to Asp in RGN1^C^) might provide a mimic continuous phosphorylation site in RGN1, thus affecting its post‐transcriptional modification or might change its protein conformation for DNA binding. Although *RGN1* ^C^ is a favourable allele mainly present in *aro* and *aus* varieties, it apparently was not selected during domestication and improvement of rice. It is possible that *RGN1* has adverse effects on other unidentified traits, or that linkage drag caused by undesirable genes in its vicinity might have constrained its selection. For example, we found that *OsETT2* affecting awn development (Toriba and Hirano, 2014) is located about 750 kb upstream of *RGN1* and elimination of the undesirable allele(s) controlling awn formation could have prevented selection of the *RGN1* ^C^ allele during domestication.

We are currently facing a severe constraint in yield potential in rice (Wei *et al*., 2015). A more detailed understanding of the mechanism of grain initiation could provide new approaches for breeders to increase yield. From this study, we have a preliminary understanding of the mechanism of how lateral grains are produced on SBs. The mechanism could also serve as a reference for maize, wheat and other cereal crops.

## Methods

### Plant materials and growth conditions

A cross between rice (*Oryza sativa*) varieties BS208 and Teqing was used to map the *RGN1* locus. NIL‐*RGN1* and NIL‐*rgn1* were homozygous sister lines with contrasting phenotypes selected from an F_7_ population derived from an F_2_ *RGN1/rgn1* heterozygote. T‐DNA insertion mutant *rgn1‐D* (PFG_3A‐03714.R) and its wild type (variety Dongjin) were purchased from the Rice T‐DNA Insertion Sequence Database. Nipponbare (NIP) and BS208 were used for transformation. All rice plants were grown under natural paddy conditions at Beijing or at Sanya in Hainan province.

### Vector constructions and plant transformation

To construct the complementation vector, RGN1‐Comp‐1F and RGN1‐Comp‐1R primers were employed to amplify the promoter of *RGN1*, and RGN1‐Comp‐2F and RGN1‐Comp‐2R primers were employed to amplify the CDS of *RGN1*. The two fragments were fused and cloned into the P*me*I and A*sc*I sites of binary plant expression vector pMDC162. To construct the CRISPR/Cas9 vector, a 20‐bp PAM sequence from the *RGN1* CDS was selected for specific recognition and cloned into vector SK‐gRNA as previously described (Wang *et al*., 2015). To construct *RGN1* and *LOG* over‐expression vectors, full‐length *RGN1* and *LOG* cDNA without stop codons were amplified by PCR and cloned into the K*pn*I and H*ind*Ⅲ sites of binary plant expression vector pC1305. To construct the *35S:Flag‐RGN1* vector, full‐length *RGN1* cDNA without a stop codon was amplified and cloned into the S*pe*I and K*pn*I sites of binary plant expression vector pCM1307. To construct the *ProRGN1:GUS* vector, a genomic fragment of the *RGN1* promoter region starting at 2.5 kb upstream of the ATG initiation codon was amplified and cloned into the P*me*I and A*sc*I sites of the binary plant expression vector pMDC162. For subcellular localization, *RGN1* cDNA and *rgn1* cDNA without stop codons were amplified by PCR with primers RGN1‐GFP‐F and RGN1‐GFP‐R and cloned into the K*pn*I and H*ind*Ⅲ sites of the binary plant expression vector pSuper1300‐GFP. To produce transgenic plants, the corresponding constructs were introduced into *Agrobacterium tumefaciens* strain EHA105 and subsequently transformed into selected rice varieties through *Agrobacterium*‐mediated transformation (Toki *et al*., 2006).

### Expression pattern analysis

qRT‐PCR was conducted to check the relative expression levels of *RGN1* and other genes involved in this study. Total RNA was extracted from fresh tissues using Trizol reagent (Invitrogen, Carlsbad, CA). All experiments were conducted using a Takara Kit as previously reported (Zhang *et al*., 2018). The rice *ubiqutin1* gene served as an internal control.

GUS‐staining assays were conducted to analyse the expression pattern of *RGN1*. Tissues from *ProRGN1: GUS* transgenic plants were collected and submerged in GUS‐staining solution as previously reported (Zhang *et al*., 2018).

### Subcellular localization

Root tips from RGN1‐GFP transgenic plants stained with 1 µg/mL DAPI (4′, 6‐diamidino‐2‐phenylin‐dole, ~30 min) were used to check the subcellular location of RGN1. *ProSuper1300:RGN1‐GFP* and *ProSuper1300:rgn1‐GFP* vectors were transiently expressed in rice protoplasts to determine the subcellular locations of RGN1 and rgn1. Fluorescence signals of GFP fusion proteins were detected and photographed under a confocal microscope (Olympus FV1000,Tokyo, Japan).

### Dual‐luciferase assay

The cDNA of *rgn1*, *RGN1* ^G^ and *RGN1* ^C^ without stop codon were amplified and cloned into the K*pn*I and P*st*I sites of the *pGreenII 62‐SK*. The resulting *rgn1‐62‐SK*, *RGN1^C^‐62‐SK* and *RGN1^G^‐62‐SK* constructs were used as effectors; empty *pGreenII 62‐SK* vector was used as control; *pGreenII0800‐ProLOG:LUC* vector was used as reporter which containing *35S:REN* served as an internal reference.

Reporters and effectors or control were transformed into rice protoplasts. Firefly luciferase (LUC) and Renilla luciferase (REN) activity levels were quantified by a Dual‐Luciferase reporter assay system (Promega, Madison, Wisconsin, USA).

### Yeast one‐hybrid assay

Full‐length *RGN1* and rgn1 cDNA were amplified and cloned into the E*co*RI and X*ho*I sites of the pB42AD vector to yield *RGN1‐AD* and *rgn1‐AD* constructs respectively. Genomic fragments of the *LOG* and *Gn1a* promoter regions starting at 2.0 kb upstream of the ATG start codons were amplified by PCR and cloned into the E*co*RI and X*ho*I sites of pLacZi2µ vector to yield reporters. RGN1‐AD was co‐transformed with the reporter or control (empty pLacZi2µ vector) into the yeast strain EGY48 through the PEG/LiAc method. Positive clones were cultured on SD/‐Ura‐Trp plates containing β‐d‐galactopyranoside to check for possible interactions between RGN1 and the reporter.

### qChIP‐PCR assays

Young panicles (1 cm in length) from *35S:Flag‐RGN1* transformants were collected and cross‐linked. ChIP assays were performed using a ChIP Assay Kit (P2078, Beyotime, Shanghai, China) following the manufacturer’s instructions. The ChIP products were used for qChIP‐PCR assays. Primers LOG‐ChIP‐qRT‐F and LOG‐ChIP‐qRT‐R were designed to amplify a 101 bp fragment from the *LOG* promoter containing the MS188 binding site in the ChIP enrichment test by qPCR.

### Electrophoretic mobility shift assay

For expression and purification of the RGN1 and rgn1 proteins in *Escherichia coli*, the cDNA of *RGN1* and *rgn1* were amplified and cloned into the E*coR*I and H*ind*Ⅲ sites of vector pMAL‐c5X to yield *MBP‐RGN1* and *MBP‐rgn1* constructs. These recombinant constructs were introduced into an *Escherichia coli Rosetta* (DE3) cell line. A PurKine™ MBP‐Tag Protein Purification Kit (Dextrin) was used to purify the proteins. A fragment of the *LOG* promoter (723–742 bp upstream of the ATG codon) containing an MS188‐binding site (ACCAAA) was selected as the probe. The mutant probe was synthesized by replacing ACCAAA with TTTTTT. Biotin‐labelled primers were synthesized and purified by Invitrogen. EMSA reactions were conducted using a LightShift Chemiluminescent EMSA kit (Thermo Scientific, Waltham, MA) following the manufacturer’s instruction. Signals were detected by a Tanon‐5200 Chemiluminescent Imaging System (Shanghai,China).

### Measurement of endogenous CKs levels

Young panicles (1 cm in length) of NIL‐*RGN1* and NIL‐*rgn1* plants were collected, ground into powder, and extracted with 1 mL methanol/water/formic acid (15 : 4 : 1, V/V/V), with three independent biological repeats per sample. The combined extracts were evaporated to dryness under a nitrogen gas stream and reconstituted in 100 μL 80% methanol (V/V). Then, CK (isopentenyladenine, tZ and cZ) contents were measured by MetWare (http://www.metware.cn/) based on the AB Sciex QTRAP4500 LC‐MS/MS platform.

### Haplotype and phylogenetic tree analyses

SNPs in 2848 cultivated accessions and 446 wild rice accessions were obtained from the rice 3 K project and a previous publication (Huang *et al*., 2012; Wang *et al*., 2018) respectively. The neighbour‐joining phylogenetic tree was constructed using MEGA 5.0 (Tamura *et al*., 2011) and then visualized and annotated using EvolView (Zhang *et al*., 2012).

### Primers

Primers used in the study are listed in Data S2.

## Conflict of interest

The authors declare that they have no competing interests.

## Author contributions

G. Li designed the research and wrote the manuscript. G. Li and B. Xu performed most of experiments. Y. Xu and Z. Wu assisted with experiments. J. Xie, X. Sun, H. Guo and X. Wang assisted with data analysis. N.U. Khan assisted with revisions for the manuscript. H. Zhang, J. Li, J. Xu and W. Wang provided technical assistance. Z. Zhang designed the research, conducted origin and evolutionary analyses, and wrote and revised the manuscript. Z. Zhang and Z. Li conceived the project, designed the research and revised the manuscript.

## Supporting information


**Figure S1** Comparison of yield related traits between TQ (Teqing) and BS208.
**Figure S2** Genome constitution of NIL‐*RGN1* and NIL‐*rgn1*.
**Figure S3** Comparison of yield related traits between NIL‐*RGN1* and NIL‐*rgn1*.
**Figure S4** Characterization of complementation plants.
**Figure S5** Characterization of *rgn1‐1* and *rgn1‐2* plants.
**Figure S6** Characterization of *RGN1* over‐expression plants.
**Figure S7** Characterization of the T‐DNA insertion plant *rgn1‐D*.
**Figure S8** Sequence alignment of RGN1 with RAX proteins from Arabidopsis.
**Figure S9** Phylogenetic analysis of RGN1 protein and homologs from in rice and other angiosperm species.
**Figure S10** The expression pattern of *RGN1* in different tissues from NIP determined by qRT‐PCR.
**Figure S11** *RGN1* participates in cytokinin metabolism.
**Figure S12** RGN1^C^ causes higher expression of *LOG*.

**Data S1** The information of SNPs used in in this study.

**Data S2** The information of primers used in this study.

## References

[pbi13702-bib-0001] Ashikari, M. , Sakakibara, H. , Lin, S. , Yamamoto, T. , Takashi, T. , Nishimura, A. , Angeles, E.R. *et al*. (2005) Cytokinin oxidase regulates rice grain production. Science, 309, 741–745. 15976269 10.1126/science.1113373

[pbi13702-bib-0002] Bai, X. , Huang, Y. , Hu, Y. , Liu, H. , Zhang, B. , Smaczniak, C. , Hu, G. *et al*. (2017) Duplication of an upstream silencer of *FZP* increases grain yield in rice. Nat. Plants, 3, 885–893. 29085070 10.1038/s41477-017-0042-4

[pbi13702-bib-0003] Gallavotti, A. , Zhao, Q. , Kyozuka, J. , Meeley, R.B. , Ritter, M.K. , Doebley, J.F. , Pe, M.E. *et al*. (2004) The role of *barren stalk1* in the architecture of maize. Nature, 432, 630–635. 15577912 10.1038/nature03148

[pbi13702-bib-0004] Galli, M. , Liu, Q. , Moss, B.L. , Malcomber, S. , Li, W. , Gaines, C. , Federici, S. *et al*. (2015) Auxin signaling modules regulate maize inflorescence architecture. Proc. Natl Acad. Sci. USA, 112, 13372–13377. 26464512 10.1073/pnas.1516473112 PMC4629326

[pbi13702-bib-0005] Graham, L.E. , Cook, M.E. and Busse, J.S. (2000) The origin of plants: body plan changes contributing to a major evolutionary radiation. Proc. Natl Acad. Sci. USA, 97, 4535–4540. 10781058 10.1073/pnas.97.9.4535 PMC34322

[pbi13702-bib-0006] Greb, T. , Clarenz, O. , Schafer, E. , Muller, D. , Herrero, R. , Schmitz, G. and Theres, K. (2003) Molecular analysis of the *LATERAL SUPPRESSOR* gene in *Arabidopsis* reveals a conserved control mechanism for axillary meristem formation. Genes Dev. 17, 1175–1187. 12730136 10.1101/gad.260703 PMC196050

[pbi13702-bib-0007] Guo, D. , Zhang, J. , Wang, X. , Han, X. , Wei, B. , Wang, J. , Li, B. *et al*. (2015) The WRKY transcription factor *WRKY71/EXB1* controls shoot branching by transcriptionally regulating *RAX* genes in *Arabidopsis* . Plant Cell, 27, 3112–3127. 26578700 10.1105/tpc.15.00829 PMC4682308

[pbi13702-bib-0008] Han, Y. , Yang, H. and Jiao, Y. (2014) Regulation of inflorescence architecture by cytokinins. Front. Plant Sci. 5, 669. 25505480 10.3389/fpls.2014.00669 PMC4241816

[pbi13702-bib-0009] Huang, X. , Kurata, N. , Wei, X. , Wang, Z.‐X. , Wang, A. , Zhao, Q. , Zhao, Y. *et al*. (2012) A map of rice genome variation reveals the origin of cultivated rice. Nature, 490, 497–501. 23034647 10.1038/nature11532 PMC7518720

[pbi13702-bib-0010] Ikeda, K. , Ito, M. , NagasawaO, N. , Kyozuka, J. and Nagato, Y. (2007) Rice *ABERRANT PANICLE ORGANIZATION 1*, encoding an F‐box protein, regulates meristem fate. Plant J. 51, 1030–1040. 17666027 10.1111/j.1365-313X.2007.03200.x

[pbi13702-bib-0011] Ikeda‐Kawakatsu, K. , Maekawa, M. , Izawa, T. , Itoh, J.I. and Nagato, Y. (2012) ABERRANT PANICLE ORGANIZATION 2/RFL, the rice ortholog of *Arabidopsis* LEAFY, suppresses the transition from inflorescence meristem to floral meristem through interaction with APO1. Plant J. 69, 168–180. 21910771 10.1111/j.1365-313X.2011.04781.x

[pbi13702-bib-0012] Ishikawa, S. , Maekawa, M. , Arite, T. , Onishi, K. , Takamure, I. and Kyozuka, J. (2005) Suppression of tiller bud activity in tillering dwarf mutants of rice. Plant Cell Physiol. 46, S192. 10.1093/pcp/pci022 15659436

[pbi13702-bib-0013] Jeifetz, D. , David‐Schwartz, R. , Borovsky, Y. and Paran, I. (2011) *CaBLIND* regulates axillary meristem initiation and transition to flowering in pepper. Planta, 234, 1227–1236. 21773792 10.1007/s00425-011-1479-8

[pbi13702-bib-0014] Jiang, L. , Liu, X. , Xiong, G. , Liu, H. , Chen, F. , Wang, L. , Meng, X. *et al*. (2013) DWARF 53 acts as a repressor of strigolactone signalling in rice. Nature, 504, 401–405. 24336200 10.1038/nature12870 PMC5802366

[pbi13702-bib-0015] Jiao, Y. , Wang, Y. , Xue, D. , Wang, J. , Yan, M. , Liu, G. , Dong, G. *et al*. (2010) Regulation of *OsSPL14* by *OsmiR156* defines ideal plant architecture in rice. Nat. Genet. 42, 541–544. 20495565 10.1038/ng.591

[pbi13702-bib-0016] Jin, J.P. , Tian, F. , Yang, D.C. , Meng, Y.Q. , Kong, L. , Luo, J.C. and Gao, G. (2017) PlantTFDB 4.0: toward a central hub for transcription factors and regulatory interactions in plants. Nucleic Acids Res. 45, D1040–D1045. 27924042 10.1093/nar/gkw982 PMC5210657

[pbi13702-bib-0017] Keller, T. , Abbott, J. , Moritz, T. and Doerner, P. (2006) *Arabidopsis REGULATOR OF AXILLARY MERISTEMS1* controls a leaf axil stem cell niche and modulates vegetative development. Plant Cell, 18, 598–611. 16473968 10.1105/tpc.105.038588 PMC1383636

[pbi13702-bib-0018] Komatsu, K. , Maekawa, M. , Ujiie, S. , Satake, Y. , Furutani, I. , Okamoto, H. , Shimamoto, K. *et al*. (2003a) *LAX* and *SPA*: major regulators of shoot branching in rice. Proc. Natl Acad. Sci. USA, 100, 11765–11770. 13130077 10.1073/pnas.1932414100 PMC208832

[pbi13702-bib-0019] Komatsu, M. , Chujo, A. , Nagato, Y. , Shimamoto, K. and Kyozuka, J. (2003b) *FRIZZY PANICLE* is required to prevent the formation of axillary meristems and to establish floral meristem identity in rice spikelets. Development, 130, 3841–3850. 12835399 10.1242/dev.00564

[pbi13702-bib-0020] Koumoto, T. , Shimada, H. , Kusano, H. , She, K.C. , Iwamoto, M. and Takano, M. (2013) Rice monoculm mutation *moc2*, which inhibits outgrowth of the second tillers, is ascribed to lack of a fructose‐1,6‐bisphosphatase. Plant Biotechnol. 30, 47–56.

[pbi13702-bib-0021] Kurakawa, T. , Ueda, N. , Maekawa, M. , Kobayashi, K. , Kojima, M. , Nagato, Y. , Sakakibara, H. *et al*. (2007) Direct control of shoot meristem activity by a cytokinin‐activating enzyme. Nature, 445, 652–655. 17287810 10.1038/nature05504

[pbi13702-bib-0022] Kwon, Y. , Yu, S.I. , Park, J.H. , Li, Y. , Han, J.H. , Alavilli, H. , Cho, J.I. *et al*. (2012) *OsREL2*, a rice TOPLESS homolog functions in axillary meristem development in rice inflorescence. Plant Biotechnol. Rep. 6, 213–224.

[pbi13702-bib-0023] Li, S. , Zhao, B. , Yuan, D. , Duan, M. , Qian, Q. , Tang, L. , Wang, B. *et al*. (2013) Rice zinc finger protein DST enhances grain production through controlling *Gn1a/OsCKX2* expression. Proc. Natl Acad. Sci. USA, 110, 3167–3172. 23382237 10.1073/pnas.1300359110 PMC3581943

[pbi13702-bib-0024] Li, X. , Qian, Q. , Fu, Z. , Wang, Y. , Xiong, G. , Zeng, D. , Wang, X. *et al*. (2003) Control of tillering in rice. Nature, 422, 618–621. 12687001 10.1038/nature01518

[pbi13702-bib-0025] McSteen, P. (2009) Hormonal regulation of branching in grasses. Plant Physiol. 149, 46–55. 19126694 10.1104/pp.108.129056 PMC2613715

[pbi13702-bib-0026] McSteen, P. , Malcomber, S. , Skirpan, A. , Lunde, C. , Wu, X.T. , Kellogg, E. and Hake, S. (2007) *barren inflorescence2* encodes a co‐ortholog of the PINOID serine/threonine kinase and is required for organogenesis during inflorescence and vegetative development in maize. Plant Physiol. 144, 1000–1011. 17449648 10.1104/pp.107.098558 PMC1914211

[pbi13702-bib-0027] Muller, D. , Schmitz, G. and Theres, K. (2006) *Blind* homologous R2R3 Myb genes control the pattern of lateral meristem initiation in *Arabidopsis* . Plant Cell, 18, 586–597. 16461581 10.1105/tpc.105.038745 PMC1383635

[pbi13702-bib-0028] Oikawa, T. and Kyozuka, J. (2009) Two‐step regulation of LAX PANICLE1 protein accumulation in axillary meristem formation in rice. Plant Cell, 21, 1095–1108. 19346465 10.1105/tpc.108.065425 PMC2685638

[pbi13702-bib-0029] O'Malley, R.C. , Huang, S.C. , Song, L. , Lewsey, M.G. , Bartlett, A. , Nery, J.R. , Galli, M. *et al*. (2016) Cistrome and epicistrome features shape the regulatory DNA landscape. Cell, 165, 1280–1292. 27203113 10.1016/j.cell.2016.04.038 PMC4907330

[pbi13702-bib-0030] Raman, S. , Greb, T. , Peaucelle, A. , Blein, T. , Laufs, P. and Theres, K. (2008) Interplay of miR164, *CUP‐SHAPED COTYLEDON* genes and *LATERAL SUPPRESSOR* controls axillary meristem formation in *Arabidopsis thaliana* . Plant J. 55, 65–76. 18346190 10.1111/j.1365-313X.2008.03483.x

[pbi13702-bib-0031] Sasaki, T. and Burr, B. (2000) International Rice Genome Sequencing Project: the effort to completely sequence the rice genome. Curr. Opin. Plant Biol. 3, 138–141. 10712951 10.1016/s1369-5266(99)00047-3

[pbi13702-bib-0032] Schmitz, G. , Tillmann, E. , Carriero, F. , Fiore, C. , Cellini, F. and Theres, K. (2002) The tomato Blind gene encodes a MYB transcription factor that controls the formation of lateral meristems. Proc. Natl Acad. Sci. USA, 99, 1064–1069. 11805344 10.1073/pnas.022516199 PMC117430

[pbi13702-bib-0033] Schumacher, K. , Schmitt, T. , Rossberg, M. , Schmitz, G. and Theres, K. (1999) The *Lateral suppressor* (*Ls*) gene of tomato encodes a new member of the VHIID protein family. Proc. Natl Acad. Sci. USA, 96, 290–295. 9874811 10.1073/pnas.96.1.290 PMC15132

[pbi13702-bib-0034] Spinelli, S.V. , Martin, A.P. , Viola, I.L. , Gonzalez, D.H. and Palatnik, J.F. (2011) A mechanistic link between *STM* and *CUC1* during *Arabidopsis* development. Plant Physiol. 156, 1894–1904. 21685178 10.1104/pp.111.177709 PMC3149926

[pbi13702-bib-0035] Stirnberg, P. , van De Sande, K. and Leyser, H.M. (2002) *MAX1* and *MAX2* control shoot lateral branching in *Arabidopsis* . Development, 129, 1131–1141. 11874909 10.1242/dev.129.5.1131

[pbi13702-bib-0036] Tabuchi, H. , Zhang, Y. , Hattori, S. , Omae, M. , Shimizu‐Sato, S. , Oikawa, T. , Qian, Q. *et al*. (2011) LAX PANICLE2 of rice encodes a novel nuclear protein and regulates the formation of axillary meristems. Plant Cell, 23, 3276–3287. 21963665 10.1105/tpc.111.088765 PMC3203427

[pbi13702-bib-0037] Takeda, T. , Suwa, Y. , Suzuki, M. , Kitano, H. , Ueguchi‐Tanaka, M. , Ashikari, M. , Matsuoka, M. *et al*. (2003) The *OsTB1* gene negatively regulates lateral branching in rice. Plant J. 33, 513–520. 12581309 10.1046/j.1365-313x.2003.01648.x

[pbi13702-bib-0038] Tamura, K. , Peterson, D. , Peterson, N. , Stecher, G. , Nei, M. and Kumar, S. (2011) MEGA5: molecular evolutionary genetics analysis using maximum likelihood, evolutionary distance, and maximum parsimony methods. Mol. Biol. Evol. 28, 2731–2739. 21546353 10.1093/molbev/msr121 PMC3203626

[pbi13702-bib-0039] Tanaka, W. , Ohmori, Y. , Ushijima, T. , Matsusaka, H. , Matsushita, T. , Kumamaru, T. , Kawano, S. *et al*. (2015) Axillary meristem formation in rice requires the WUSCHEL ortholog TILLERS ABSENT1. Plant Cell, 27, 1173–1184. 25841039 10.1105/tpc.15.00074 PMC4558701

[pbi13702-bib-0040] Toki, S. , Hara, N. , Ono, K. , Onodera, H. , Tagiri, A. , Oka, S. and Tanaka, H. (2006) Early infection of scutellum tissue with *Agrobacterium* allows high‐speed transformation of rice. Plant J. 47, 969–976. 16961734 10.1111/j.1365-313X.2006.02836.x

[pbi13702-bib-0041] Toriba, T. and Hirano, H.Y. (2014) The *DROOPING LEAF* and *OsETTIN2* genes promote awn development in rice. Plant J. 77, 616–626. 24330191 10.1111/tpj.12411

[pbi13702-bib-0042] Wang, C. , Shen, L. , Fu, Y.P. , Yan, C.J. and Wang, K.J. (2015) A simple CRISPR/Cas9 system for multiplex genome editing in rice. J. Genet. Genomics, 42, 703–706. 26743988 10.1016/j.jgg.2015.09.011

[pbi13702-bib-0043] Wang, W. , Mauleon, R. , Hu, Z. , Chebotarov, D. , Tai, S. , Wu, Z. , Li, M. *et al*. (2018) Genomic variation in 3,010 diverse accessions of Asian cultivated rice. Nature, 557, 43–49. 29695866 10.1038/s41586-018-0063-9 PMC6784863

[pbi13702-bib-0044] Wei, X. , Zhang, Z. , Shi, P.J. , Wang, P. , Chen, Y. , Song, X. and Tao, F.L. (2015) Is yield increase sufficient to achieve food security in China? PLoS ONE, 10, 2. 10.1371/journal.pone.0116430 PMC4332688 25680193

[pbi13702-bib-0045] Wu, Y. , Wang, Y. , Mi, X.F. , Shan, J.X. , Li, X.M. , Xu, J.L. and Lin, H.X. (2016) The QTL *GNP1* encodes GA20ox1, which increases grain number and yield by increasing cytokinin activity in rice panicle meristems. PLoS Genet. 12, 10. 10.1371/journal.pgen.1006386 PMC5072697 27764111

[pbi13702-bib-0046] Yang, F. , Wang, Q. , Schmitz, G. , Muller, D. and Theres, K. (2012) The bHLH protein ROX acts in concert with RAX1 and LAS to modulate axillary meristem formation in *Arabidopsis* . Plant J. 71, 61–70. 22372440 10.1111/j.1365-313X.2012.04970.x

[pbi13702-bib-0047] Yao, H. , Skirpan, A. , Wardell, B. , Matthes, M.S. , Best, N.B. , McCubbin, T. , Durbak, A. *et al*. (2019) The *barren stalk2* gene is required for axillary meristem development in maize. Mol. Plant, 12, 374–389. 30690173 10.1016/j.molp.2018.12.024

[pbi13702-bib-0048] Yoshida, A. , Sasao, M. , Yasuno, N. , Takagi, K. , Daimon, Y. , Chen, R. , Yamazaki, R. *et al*. (2013) *TAWAWA1*, a regulator of rice inflorescence architecture, functions through the suppression of meristem phase transition. Proc. Natl Acad. Sci. USA, 110, 767–772. 23267064 10.1073/pnas.1216151110 PMC3545824

[pbi13702-bib-0049] Zhang, H. , Gao, S. , Lercher, M.J. , Hu, S. and Chen, W.H. (2012) EvolView, an online tool for visualizing, annotating and managing phylogenetic trees. Nucleic Acids Res. 40, W569–W572. 22695796 10.1093/nar/gks576 PMC3394307

[pbi13702-bib-0050] Zhang, Z. , Li, J. , Yao, G. , Zhang, H. , Dou, H. , Shi, H. , Sun, X. *et al*. (2011) Fine mapping and cloning of the *Grain Number Per‐Panicle Gene (Gnp4)* on chromosome 4 in rice *(Oryza sativa L.)* . Agric. Sci. China, 10, 1825–1833.

[pbi13702-bib-0051] Zhang, Z. , Li, J. , Tang, Z. , Sun, X. , Zhang, H. , Yu, J. , Yao, G. *et al*. (2018) Gnp4/LAX2, a RAWUL protein, interferes with the OsIAA3‐OsARF25 interaction to regulate grain length via the auxin signaling pathway in rice. J. Exp. Bot. 69, 4723–4737. 30295905 10.1093/jxb/ery256 PMC6137978

[pbi13702-bib-0052] Zhou, F. , Lin, Q. , Zhu, L. , Ren, Y. , Zhou, K. , Shabek, N. , Wu, F. *et al*. (2013) D14‐SCF(D3)‐dependent degradation of D53 regulates strigolactone signalling. Nature, 504, 406–410. 24336215 10.1038/nature12878 PMC4096652

