# Supplementary material for: RGN1 controls grain number and shapes panicle architecture in rice
Source: Plant Biotechnol J. 2021 Sep 22;20(1):158–67. doi: 10.1111/pbi.13702 (PMC8710824; doi:10.1111/pbi.13702)
Supplement: Supplementary file 1 — Figure S1 Comparison of yield related traits between TQ (Teqing) and BS208. Figure S2 Genome constitution of NIL‐RGN1 and NIL‐rgn1. Figure S3 Comparison of yield related traits between NIL‐RGN1 and NIL‐rgn1. Figure S4 Characterization of complementation plants. Figure S5 Characterization of rgn1‐1 and rgn1‐2 plants. Figure S6 Characterization of RGN1 over‐expression plants. Figure S7 Characterization of the T‐DNA insertion plant rgn1‐D. Figure S8 Sequence alignment of RGN1 with RAX proteins from Arabidopsis. Figure S9 Phylogenetic analysis of RGN1 protein and homologs from in rice and other angiosperm species. Figure S10 The expression pattern of RGN1 in different tissues from NIP determined by qRT‐PCR. Figure S11 RGN1 participates in cytokinin metabolism. Figure S12 RGN1C causes higher expression of LOG. [file PBI-20-158-s003.docx]

# *Supplementary figures for*

# *RGN1* controls grain number and shapes panicle architecture in rice

Gangling Li^1,†^,^，^Bingxia Xu^1,†^, Yanpei Zhang^1^, Yawen Xu^1^, Najeeb Ullah Khan^1^, Jianyin Xie^1^, Xingming Sun^1^, Haifeng Guo^1^, Zhenyuan Wu^1^, Xueqiang Wang^1^, Hongliang Zhang^1^, Jinjie Li^1^, Jianlong Xu^2^, Wensheng Wang^2^, Zhanying Zhang^1, *^, Zichao Li^1, *^

1. State Key Laboratory of Agrobiotechnology / Beijing Key Laboratory of Crop Genetic Improvement, College of Agronomy and Biotechnology, China Agricultural University, Beijing 100193, China.

2. Institute of Crop Sciences, Chinese Academy of Agricultural Sciences, Beijing 100081, China.

^†^. These authors contributed equally to this work.

*. corresponding author: [zhangzhanying@cau.edu.cn](mailto:zhangzhanying@cau.edu.cn); lizichao@cau.edu.cn

**
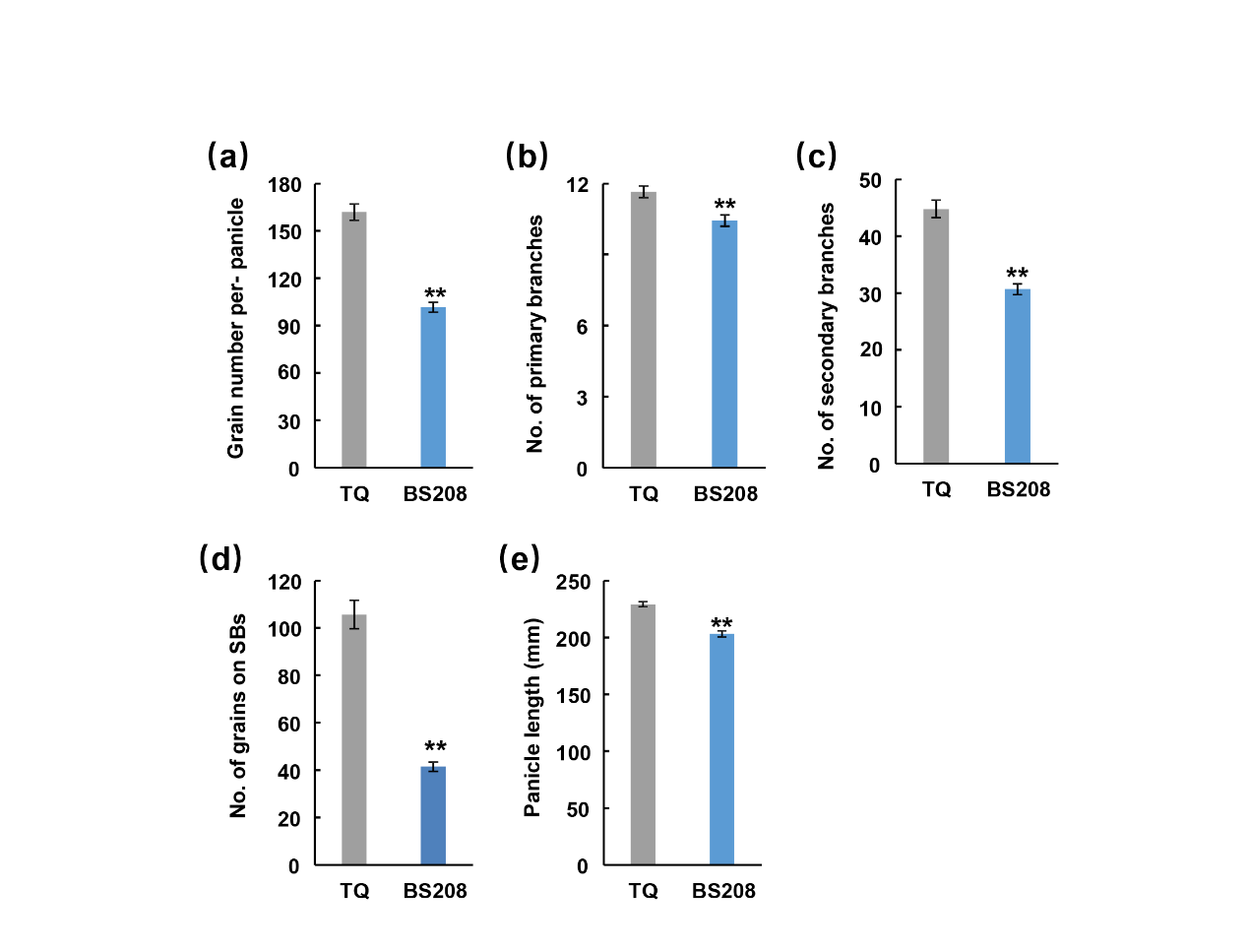
**

**Figure S1. Comparison of yield related traits between TQ (Teqing) and BS208**

SB, secondary branch. Values are means ± S.E.M., (n = 15). **, P <0.01, Two-tailed student's *t*-tests.


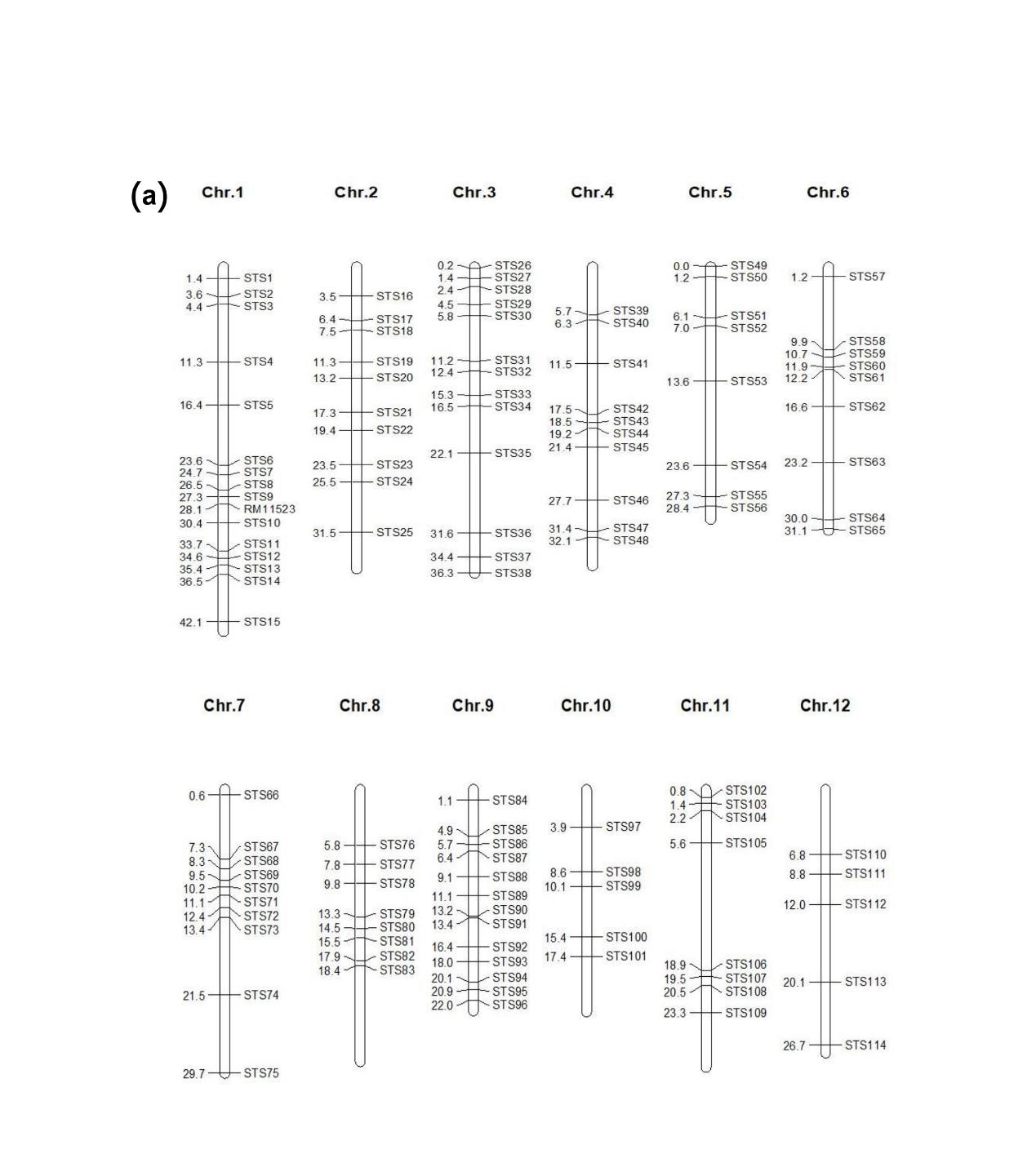


**(Continued)**


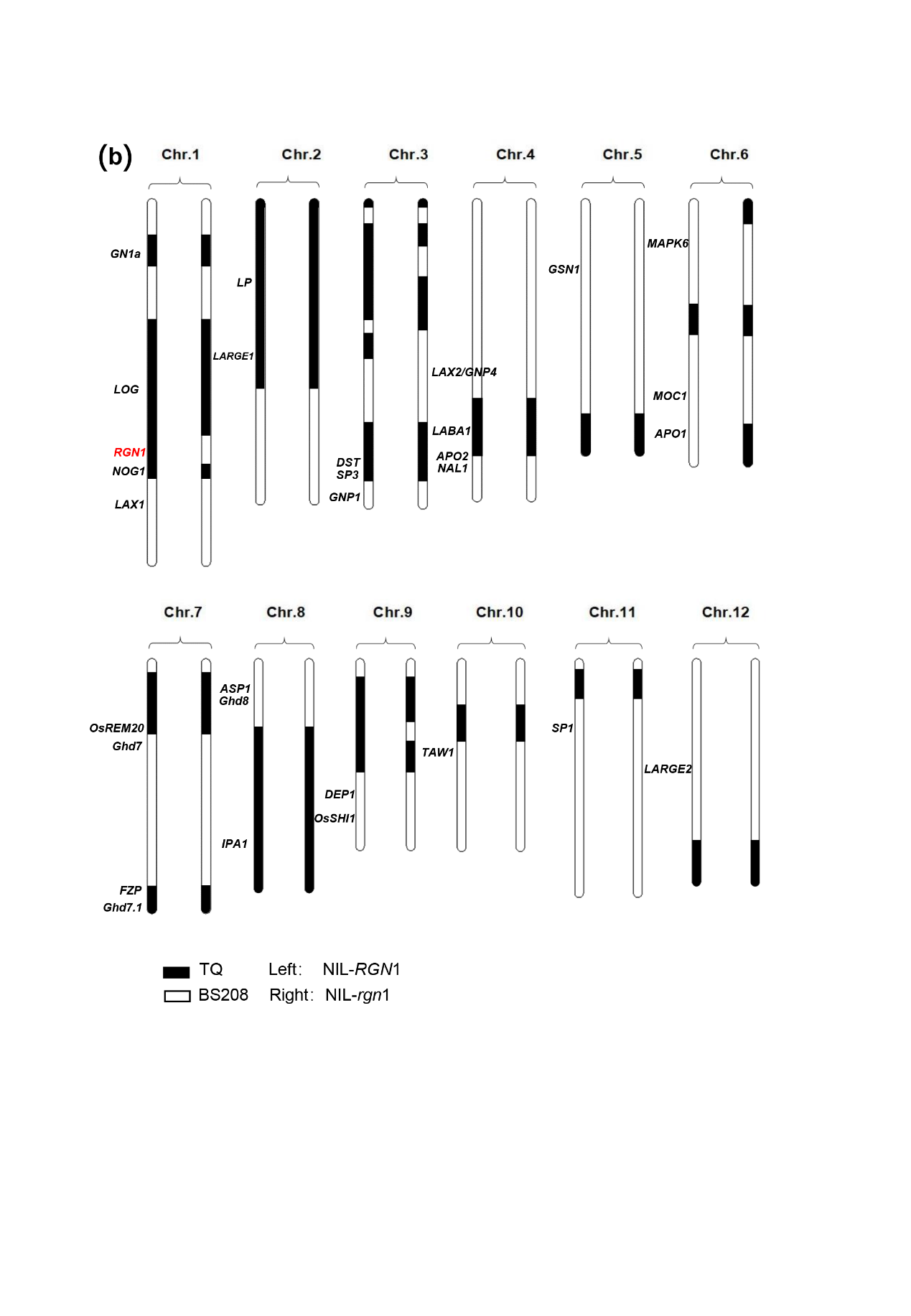


**Figure S2. Genome constitution of NIL-*RGN1* and NIL-*rgn1***

(**a**) 115 polymorphism markers were used for genotyping of NIL-*RGN1* and NIL-*rgn1*. (**b**) Graphical genotypes of NIL-*RGN1* and NIL-*rgn1*. The black regions indicate segments from TQ, and the white regions indicate segments from BS208. Cloned genes conferring grain number per-panicle were indicated on the left of sketch map for NIL-*RGN1*. The NILs differed at seven genomic locations. One difference on Chromosome 6 involved *ABERRANT PANICLE ORGANIZATION1* (*APO1*) which determines panicle length.


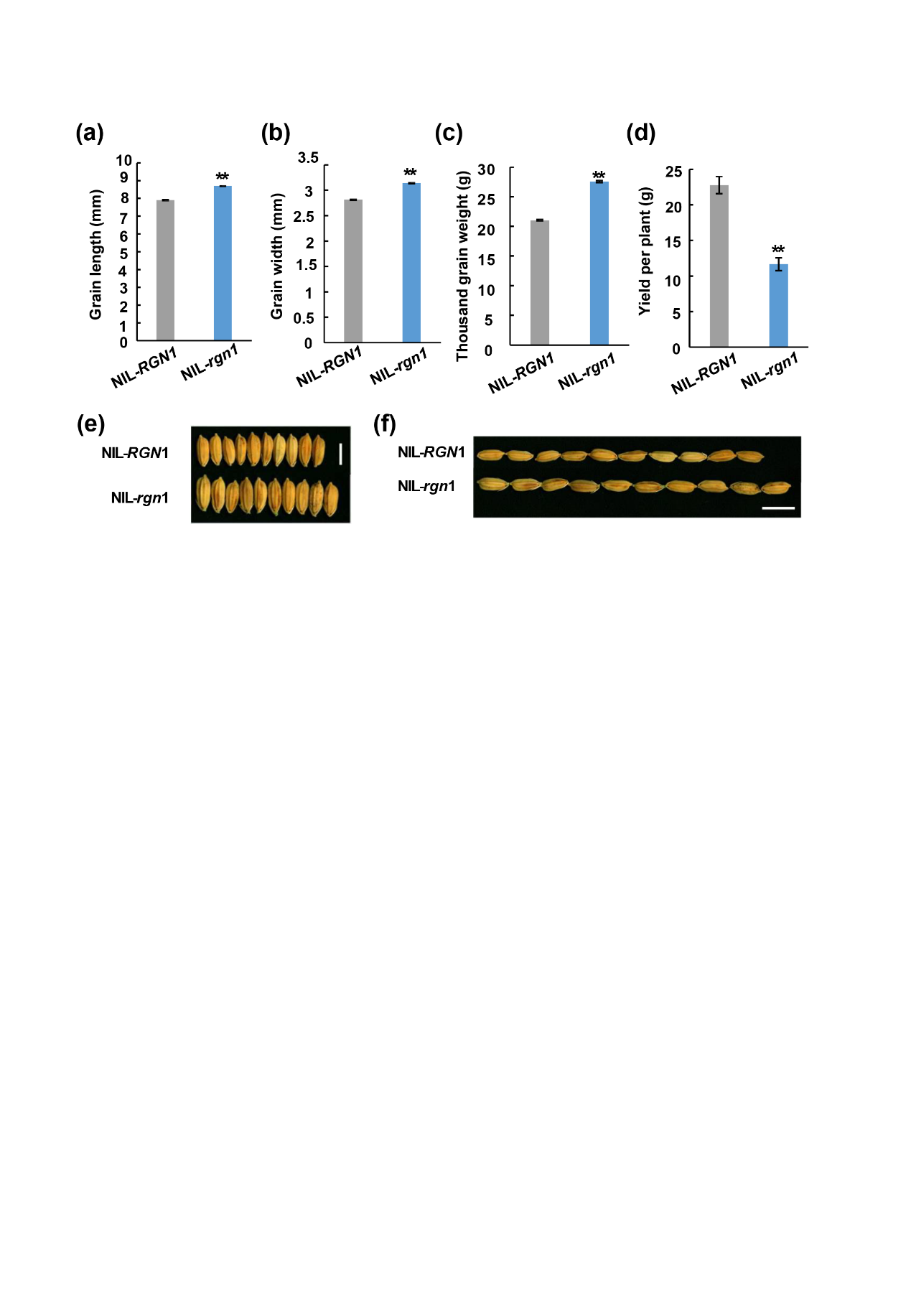


**Figure S3. Comparison of yield related traits between NIL-*RGN1* and NIL-*rgn1***

Scale bars, 5 mm for (e); 1 cm for (f). Values are means ± S.E.M., (n = 19). **, P <0.01, Two-tailed student's *t*-tests.


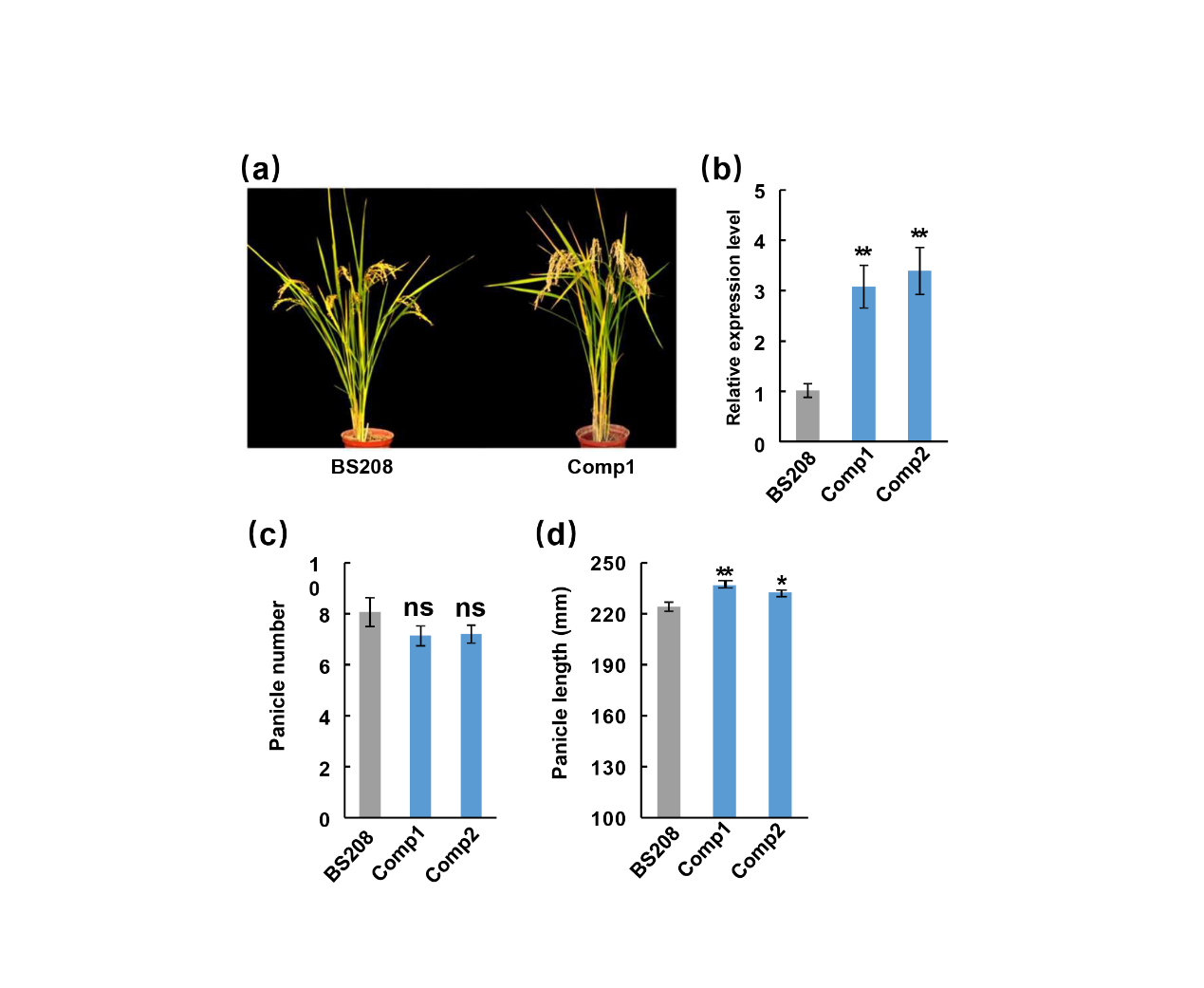


**Figure S4. Characterization of complementation plants**

(**a**) Plant architecture of BS208 and Comp1 plants. (**b**) Expression level of *RGN1*. Values are means ± S.E.M., (n = 3 plants, each with three technical repeats). (**c-d**) Statistical results for panicle number (c) and panicle length (d) of BS208, Comp1 and Comp2 plants. Values are means ± S.E.M., (n = 15). *, P <0.05; **, P <0.01, Two-tailed student's *t*-tests.


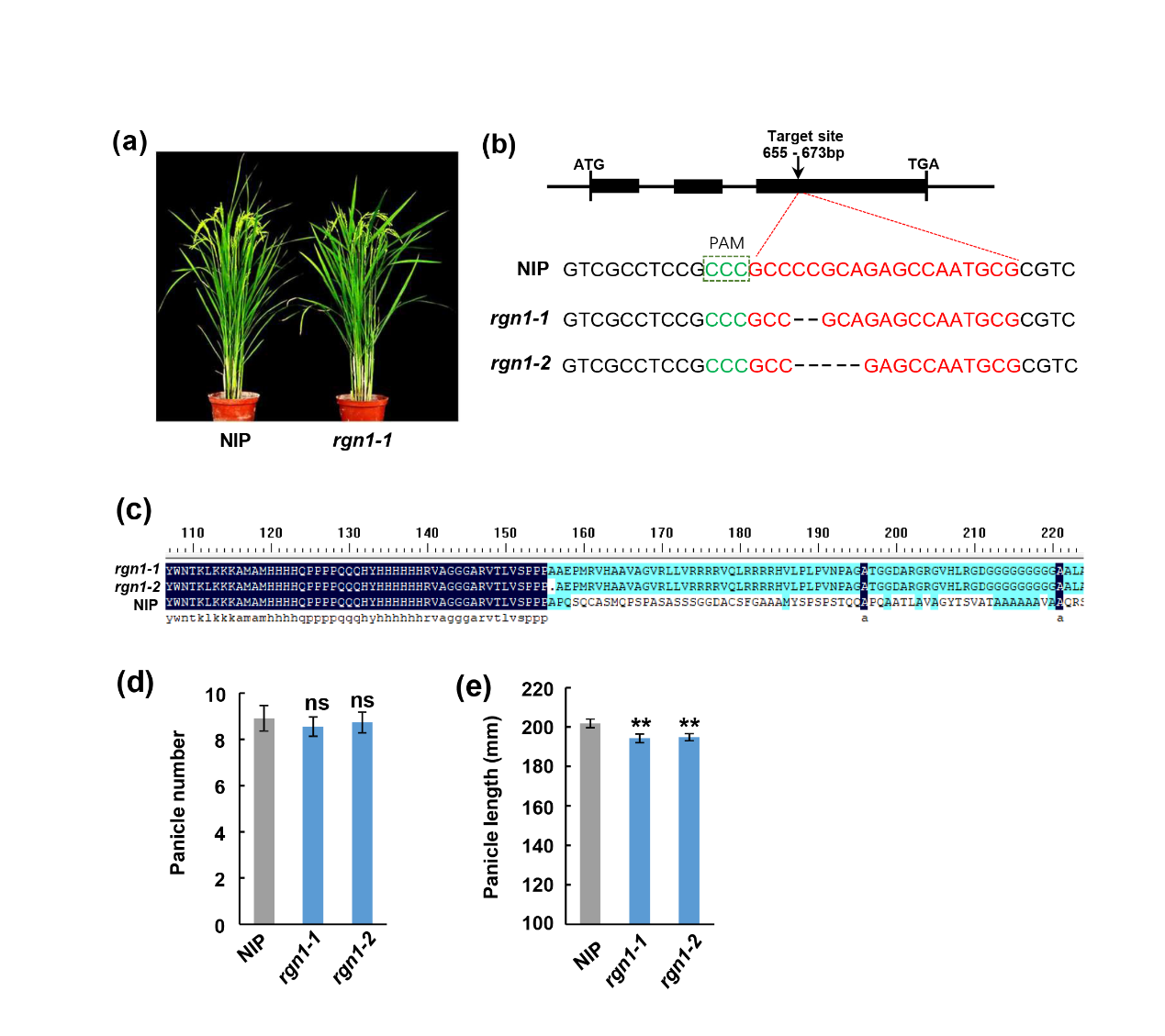


**Figure S5. Characterization of *rgn1-1* and *rgn1-2*** **plants**

(**a**) Plant architecture of *rgn1-1* plants. (**b**) Mutation of *RGN1* in *rgn1-1* and *rgn1-2* plants. Target site in the third exon is indicated by black arrow in the *RGN1* gene model, the target sequence is in red script, and protospacer-adjacent motif (PAM) sequence is indicated by green letters. Black dashes represent deletions. (**c**) Alignment of the amino acid residues of RGN1 from NIP, *rgn1-1* and *rgn1-2*. (**d-e**) Statistical results for panicle number (c) and panicle length (d) of NIP, *rgn1-1* and *rgn1-2* plants. Values are means ± S.E.M., (n = 12). *, P <0.05; **, P <0.01, Two-tailed student's *t*-tests.


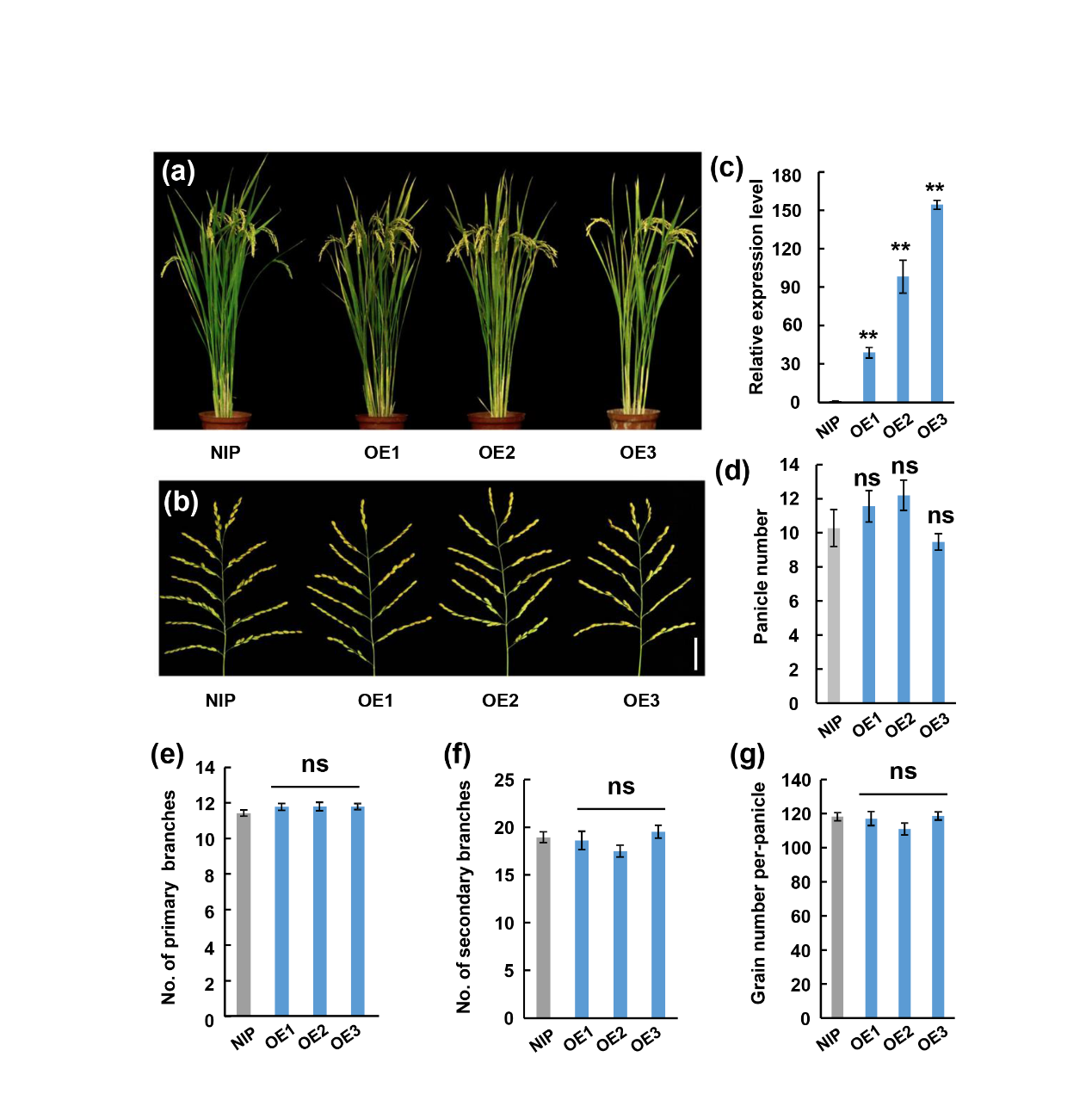


**Figure S6. Characterization of *RGN1* overexpression plants**

(**a-b**) Plant architectures (a) and panicles (b) of NIP and *RGN1* overexpression plants. (**c**) *RGN1* expression level in NIP and *RGN1* overexpression plants. Values are means ± S.E.M., (n = 3 plants, each with three technical repeats). (**d-g**) Comparisons of panicle number (d), primary branch number (e), secondary branch number (f), and grain number per-panicle (g) between NIP and *RGN1* overexpression plants. Values are means ± S.E.M., (n = 12). **, P <0.01, Two-tailed student's *t*-tests.


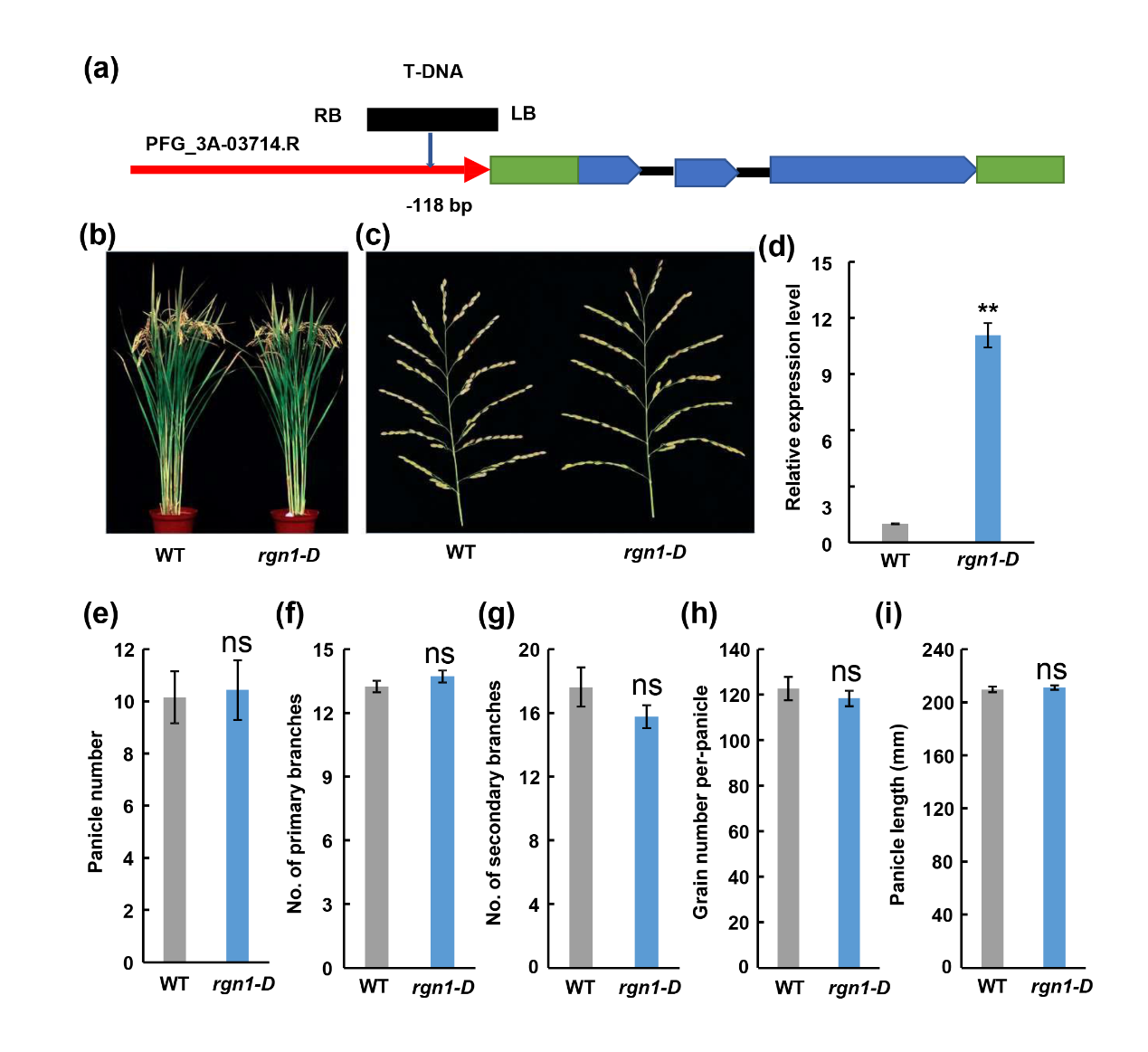


**Figure S7. Characterization of the T-DNA insertion plant *rgn1-D***

(**a**) Schematic representation of the T-DNA insertion site in the promoter region of *RGN1*. LB, T-DNA left border; RB, T-DNA right border. (**b-c**) The plant architecture (b) and panicles (c) of WT and *rgn1-D* plants. WT represents wild type Dongjin. (**d**) *RGN1* expression level in WT and *rgn1-D* plants. Values are means ± S.E.M., (n = 3 plants, each with three technical repeats). (**e-i**) Comparisons of panicle number(e), primary branches number(f), second branch number(g), grain number per-panicle(h), and panicle length(i) between of WT and *rgn1-D* plants. Values are means ± S.E.M., (n = 14). **, P <0.01, Two-tailed student's *t*-tests.


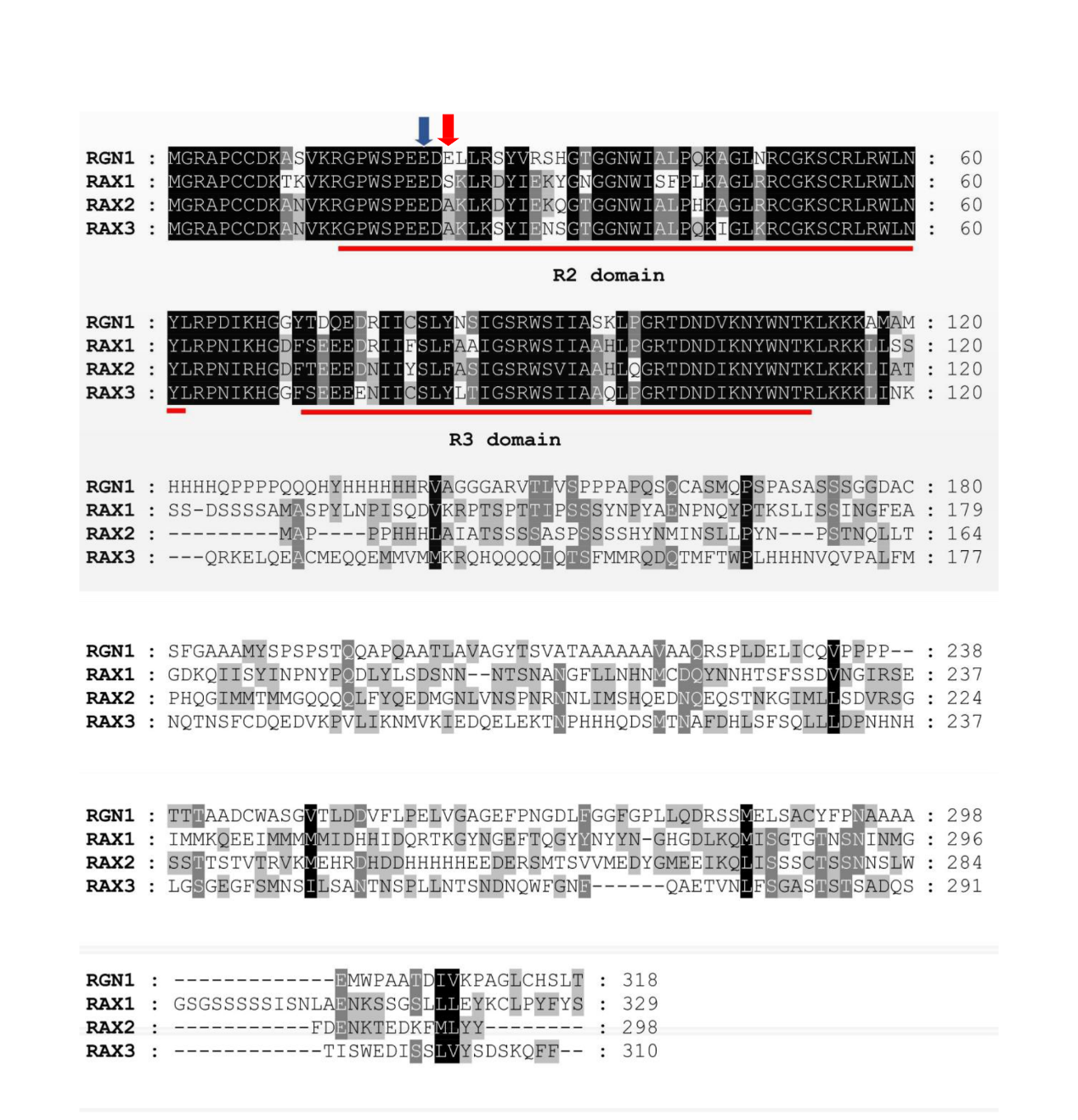


**Figure S8. Sequence alignment of RGN1 with RAX proteins from Arabidopsis**

The conserved R2R3 DNA binding domain is indicated by red underlining. The blue arrow points to the amino acid that is missing in BS208 and the red arrow points to the amino acid change caused by the non-synonymous mutation S28250218.


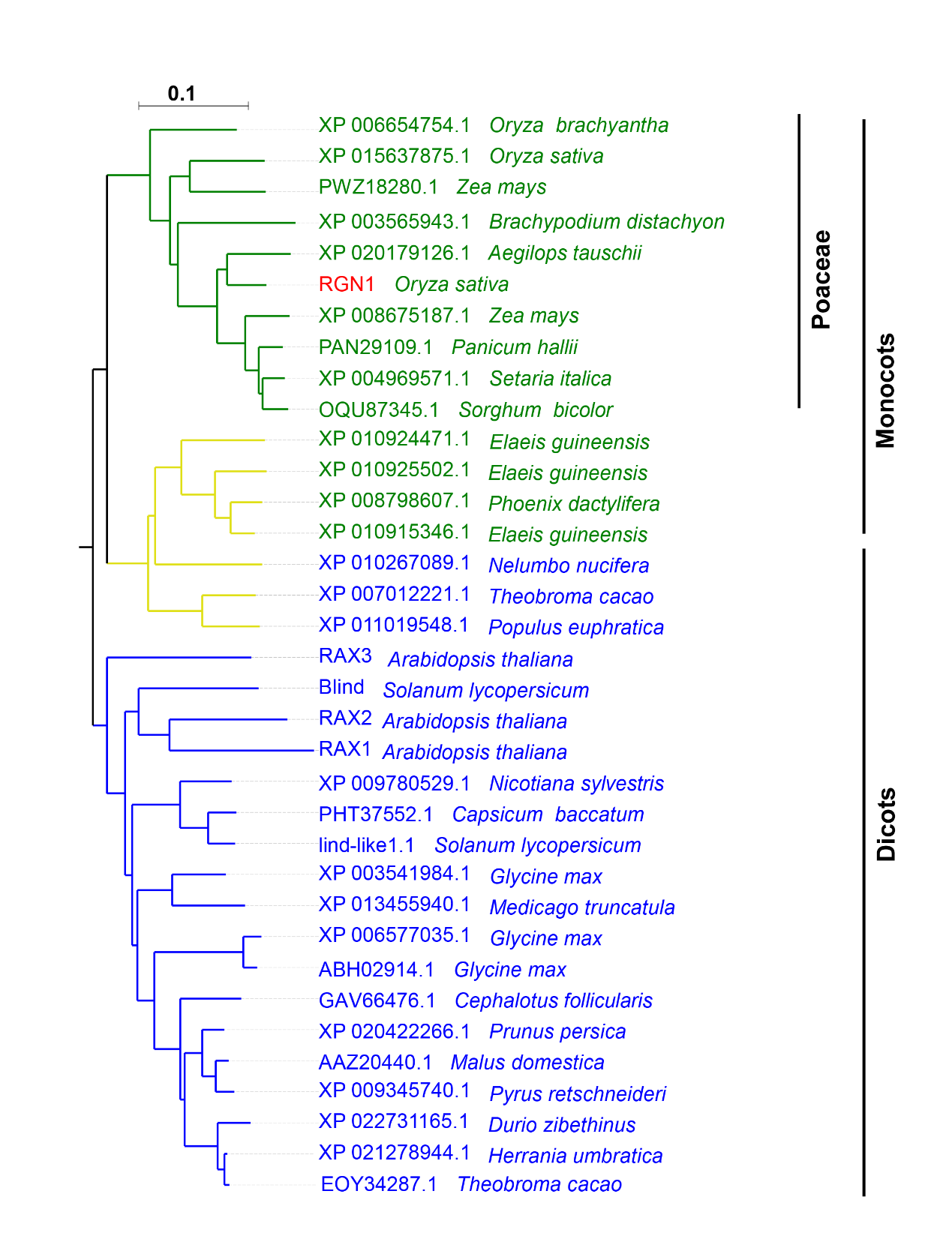


**Figure S9. Phylogenetic analysis of RGN1 protein and homologs from in rice and other angiosperm species**

The phylogenetic tree was constructed by MEGA 5 using the Neighbor-Joining method. Scale bar represents the evolutionary distances computed using the Jones-Taylor- Thomton (JTT) model.


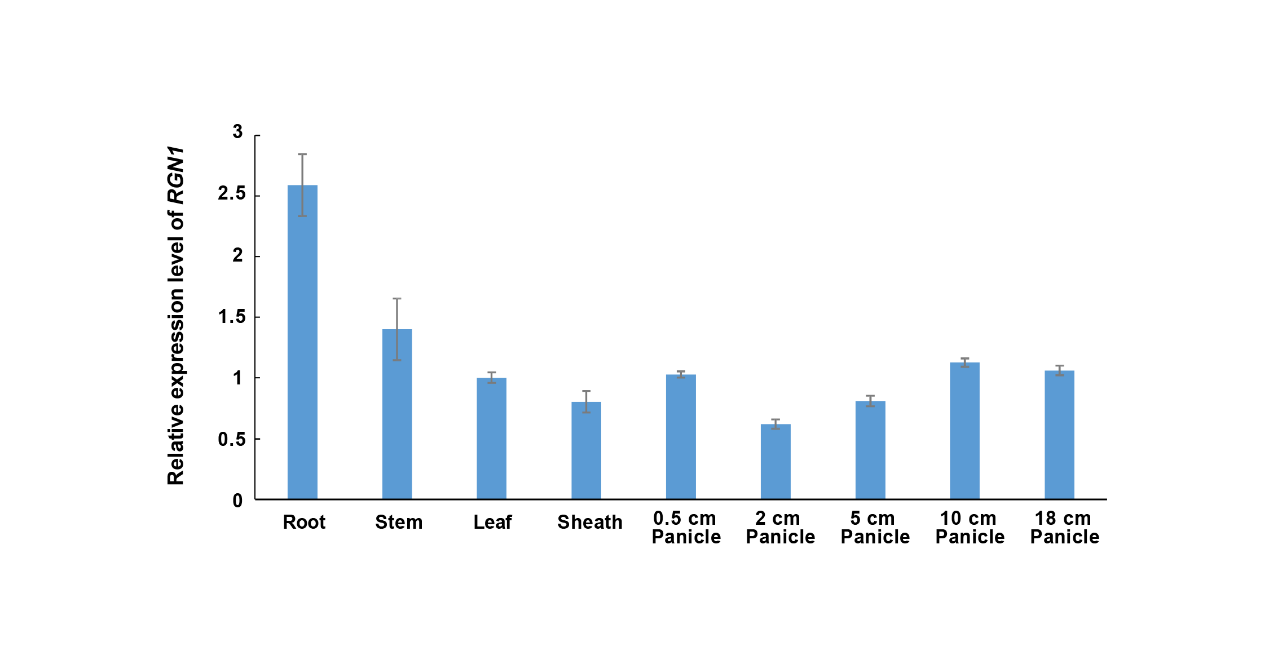


**Figure S10. The expression pattern of *RGN1* in different tissues from NIP determined by qRT-PCR.**

Values are means ± S.E.M., (n = 3 plants, each with three technical repeats).


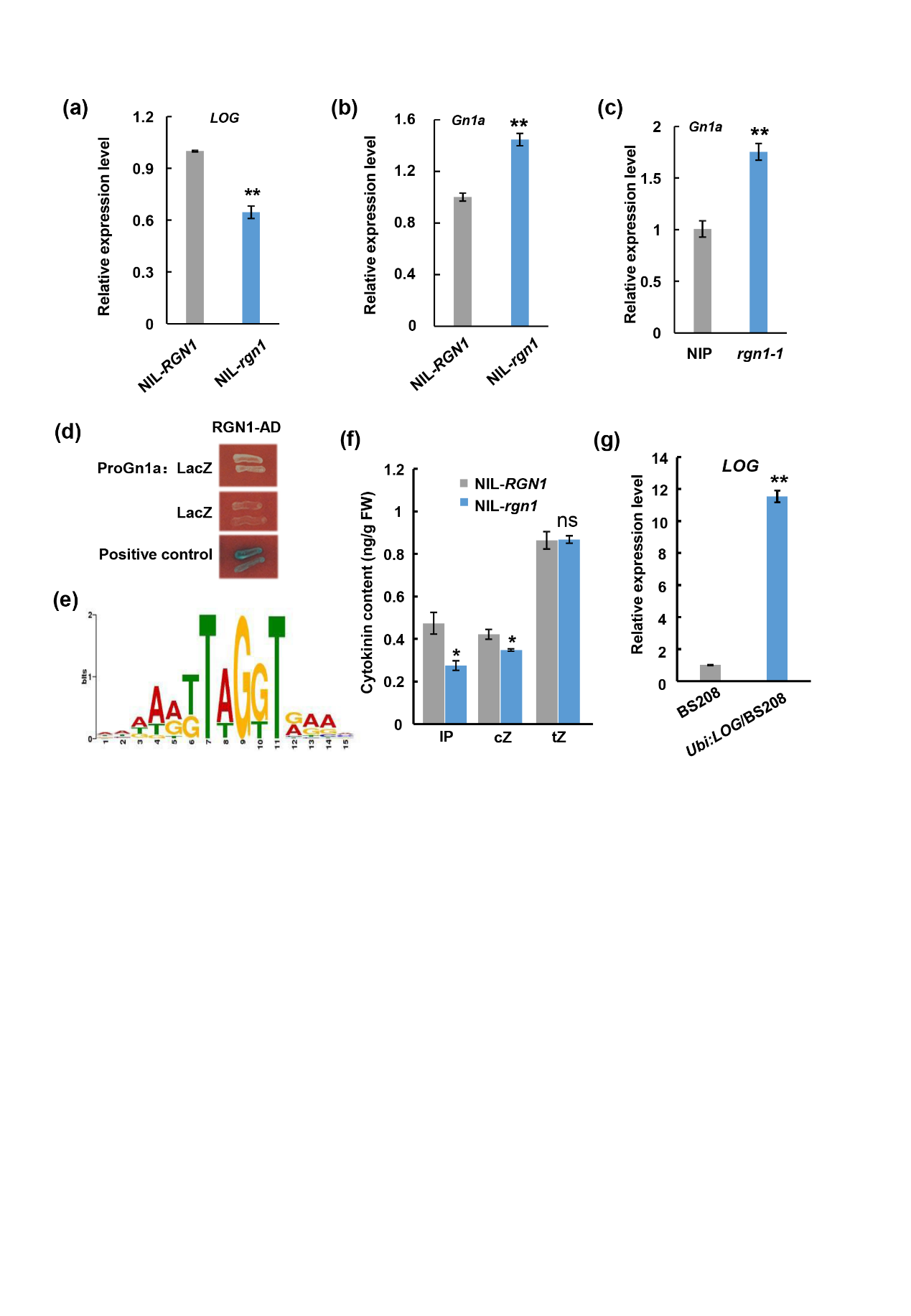


**Figure S11. *RGN1* participates in cytokinin metabolism**

(**a-b**) Expression levels of *LOG* and *Gn1a* in NIL-*RGN1 and* NIL-*rgn1*. (**c**) Expression levels of *Gn1a* in NIP and *rgn1-1* plant. (**d**) Yeast one-hybrid assays (Y1H) showing that RGN1 cannot bind to the promoter of *Gn1a*. The ProOsERF142:LacZ reporter and pB42AD-OsARF25 were used as positive controls. (**e**) The MS188 binding motif identified from PlantTFDB. (**f**) Comparison of CKs contents in young panicles between NIL-*RGN1* and NIL-*rgn1*. (**g**) *LOG* expression level in BS208 and *LOG* overexpression line. Values are means ± S.E.M., (n = 3). *, P <0.05, **, P <0.01, Two-tailed student's *t*-tests. FW, fresh weight.


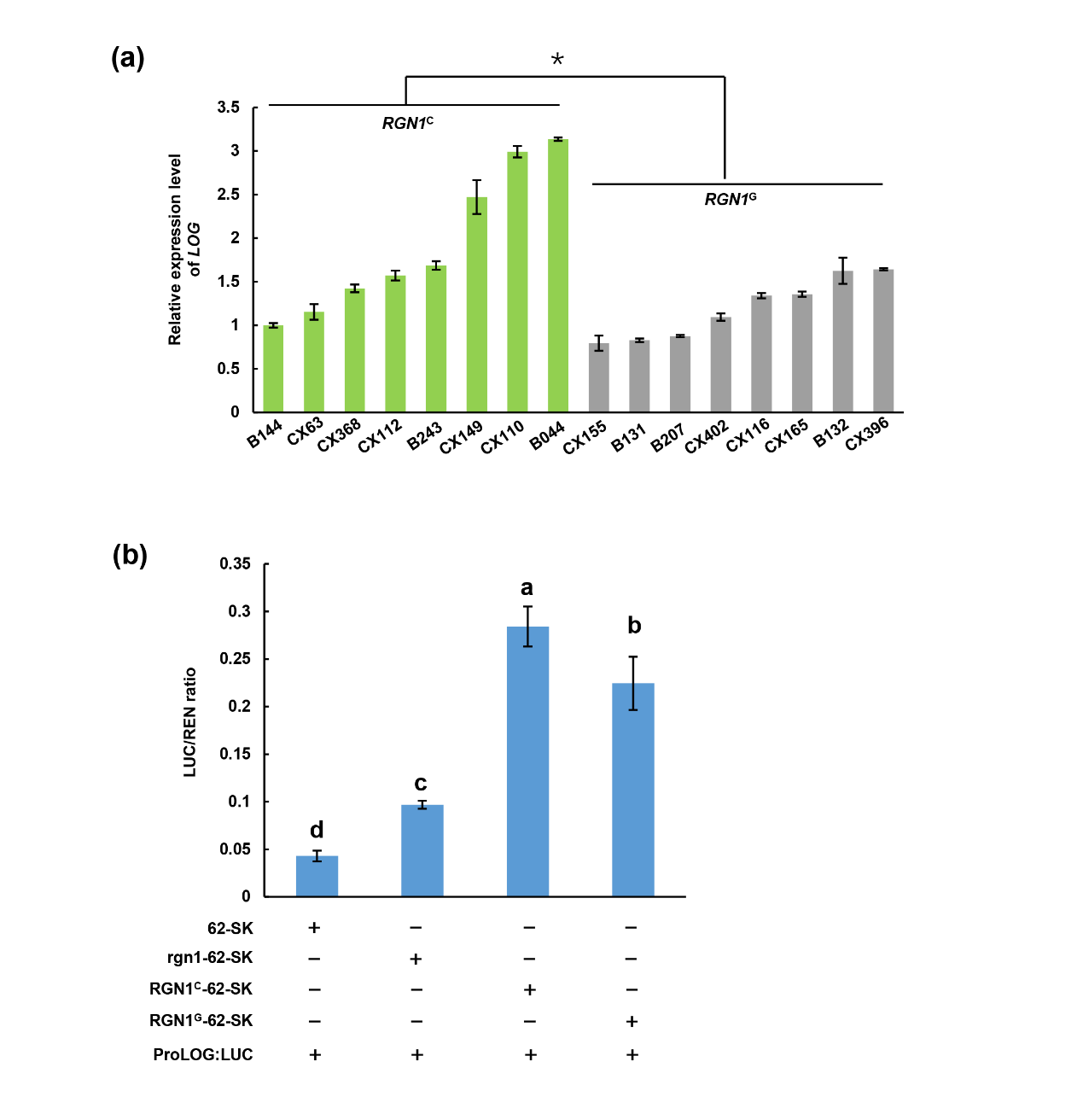


**Figure S12. RGN1^C^ causes higher expression of *LOG***

(**a**) Rice accessions harboring the *RGN1*^C^ allele caused higher expression level of *LOG.*  Values are means ± S.E.M., (n = 3). *, P <0.05, Two-tailed student's *t*-test. (**b**) Dual luciferase assay showing RGN1^C^ has stronger effect on the expression of *LOG* in rice protoplasts. The presence of the same lowercase letter above the error bar denotes a non-significant difference between the means (P >0.05, Student’s *t*-tests).
